# Adipose-Derived Stem Cells Spontaneously Express Neural Markers When Grown in a PEG-Based 3D Matrix

**DOI:** 10.3390/ijms241512139

**Published:** 2023-07-28

**Authors:** Neus Gomila Pelegri, Aleksandra M. Stanczak, Amy L. Bottomley, Bruce K. Milthorpe, Catherine A. Gorrie, Matthew P. Padula, Jerran Santos

**Affiliations:** 1 Advanced Tissue Engineering and Stem Cell Biology Group, School of Life Sciences, University of Technology Sydney, Ultimo, NSW 2007, Australia; neus.gomilapelegri@uts.edu.au (N.G.P.); bruce.milthorpe@uts.edu.au (B.K.M.); 2 Neural Injury Research Unit, School of Life Sciences, University of Technology Sydney, Ultimo, NSW 2007, Australia; catherine.gorrie@uts.edu.au; 3 School of Life Sciences, University of Technology Sydney, Ultimo, NSW 2007, Australia; aleksandra.m.stanczak@student.uts.edu.au (A.M.S.); matthew.padula@uts.edu.au (M.P.P.); 4 Microbial Imaging Facility, University of Technology Sydney, Ultimo, NSW 2007, Australia; amy.bottomley@uts.edu.au

**Keywords:** tissue engineering, bioprinting, polyethylene glycol, PEG, neural differentiation, adipose-derived stem cells, hydrogels, immunocytochemistry, CNPase, proteomics

## Abstract

Neurological diseases are among the leading causes of disability and death worldwide and remain difficult to treat. Tissue engineering offers avenues to test potential treatments; however, the development of biologically accurate models of brain tissues remains challenging. Given their neurogenic potential and availability, adipose-derived stem cells (ADSCs) are of interest for creating neural models. While progress has been made in differentiating ADSCs into neural cells, their differentiation in 3D environments, which are more representative of the in vivo physiological conditions of the nervous system, is crucial. This can be achieved by modulating the 3D matrix composition and stiffness. Human ADSCs were cultured for 14 days in a 1.1 kPa polyethylene glycol-based 3D hydrogel matrix to assess effects on cell morphology, cell viability, proteome changes and spontaneous neural differentiation. Results showed that cells continued to proliferate over the 14-day period and presented a different morphology to 2D cultures, with the cells elongating and aligning with one another. The proteome analysis revealed 439 proteins changed in abundance by >1.5 fold. Cyclic nucleotide 3′-phosphodiesterase (CNPase) markers were identified using immunocytochemistry and confirmed with proteomics. Findings indicate that ADSCs spontaneously increase neural marker expression when grown in an environment with similar mechanical properties to the central nervous system.

## 1. Introduction

Neurological disorders affect the body’s peripheral and central nervous systems [1] and are among the leading causes of disability and death worldwide [2]. For most nervous system disorders, such as spinal cord injury, traumatic brain injury, stroke, multiple sclerosis and Alzheimer’s disease, cures are unavailable, and treatment remains complex and can result in morbidity as well as significant social and economic impact [1,2,3].

Significant effort has been made to further understand and treat these and other neurological disorders; however, the gold standard in such research involves animal models, ex vivo samples or 2D cell culture models. Results from such studies typically translate poorly from animal models to the clinic, which has been attributed to the lack of accurate in vitro models of the nervous system [4,5,6].

**Figure 1:**
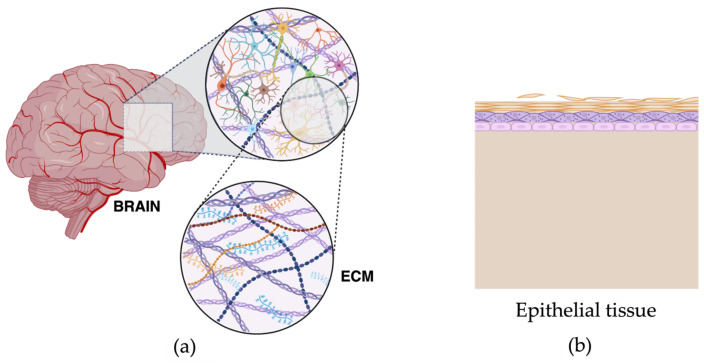
Diagrams showing the complex 3D brain structure versus squamous epithelium that might be modelled by 2D cell culture. (**a**) Brain structure and composition showing different cell types including glia, neurons, astrocytes, microglia, oligodendrocytes and other support cells. It also depicts the ECM and the different components of brain ECM like laminins, proteoglycans, collagen and hyaluronic acid. (**b**) Structure and composition of simple epithelial tissue with epithelial cells and basal membrane. Created with BioRender.com, accessed 23 June 2023.

The human brain is one of the most complex biological systems and, as such, is poorly replicated in animal models as well as in in vitro systems. Contrary to other simpler organs, the cellular organisation and structure of the brain contains a high density of many cell types with diverse synaptic connections and interactions forming large and complex neural circuits. It is composed of cells like neurons, oligodendrocytes, glial cells, astrocytes, and other support cells, as well as an extracellular matrix (ECM). The brain ECM represents 20–30% of the brain occupying the space between neural cells and mainly formed of glycosaminoglycans like hyaluronic acid, proteoglycans like neurocan, glycoproteins like tenascin-R and fibrous proteins like collagen and fibronectin (Figure 1) [4,5,7,8,9,10]. The ECM is a physical barrier that assists in diffusion within the brain and has a role in neural development, including neurite outgrowth, synaptogenesis and plasticity [11,12,13]. Additionally, the brain is the softest tissue in the body, with an elastic modulus that ranges from 0.1 kPa to 1.6 kPa [14,15], making it even more challenging to replicate in vitro. Figure 1 shows a visual representation of the differences between brain structure compared to the epithelial tissue that lines different organs like lungs.

There are considerable challenges in sourcing healthy human brain tissue or cells for experimental research due to the physical inaccessibility and high risk of damage, which makes it difficult to investigate the normal properties and behaviours of these cells in vitro. Without a complete understanding of the pathophysiology of neurological conditions, it is challenging to identify and validate potential therapeutic targets [5].

Adipose-derived stem cells (ADSCs) are an ideal candidate to develop disease models, as well as investigating potential treatment avenues due to their neurogenic potential, abundance and accessibility [16,17]. Not only are they highly abundant in the human body, they are also relatively easily accessible through subcutaneous adipose tissue liposuction, minimizing potential collection complications from more invasive cell collection methods like bone marrow aspirates [18]. Additionally, significant progress has been made in differentiating ADSCs toward neural cells; however, the most common method of differentiation is with 2D chemical inductions, which are not always stable [19,20,21,22,23,24,25,26]. Ahmadi et al. [27] compared the stability of ADSCs neural differentiation between a 2D chemical induction protocol and a sphere formation protocol. The results showed that while treated ADSCs from both protocols had large expression of neural-specific markers, and 2D chemical induction showed a rapid differentiation, it also led to further cell death and the neural-like state appeared to be reversible. On the other hand, the neurosphere formation protocol was slower, it had better cell viability and the neural-like state was more stable [27]. Differentiation in 3D matrices that are more representative of the natural physiology of the nervous system may present benefits and yield higher translation to the clinic.

Tissue engineering offers a potential avenue to create a more accurate/representative in vitro model and take into consideration the effects of environmental and mechanical cues on cell differentiation [28,29].

There is increasing evidence that cells grown in 3D conformations show responses more comparable to in vivo behaviours while varying considerably both morphologically and physiologically from cells grown in 2D monolayers. In 3D culture models, cells replicate the shape and organization found in tissue as cells are allowed to grow in aggregates or spheroids containing multiple layers [28,30,31,32,33,34]. Additionally, cell exposure to nutrients and waste is also closer to in vivo conditions, where nutrients and growth factors/drug treatments are not equally distributed among all cells, which means cells are often more resistant to drug treatments and have higher rates of resistance to drug-induced apoptosis providing a more accurate prediction of in vivo drug response [28,33,35,36,37,38,39]. Furthermore, cell proliferation is also more realistic in 3D cultures compared to the unnatural rapid pace at which cells grow in 2D [34,35,36,40], while gene expression and protein abundance better resemble levels observed in in vivo tissues [33,34,40]. Cells also respond more accurately to the mechanical stimuli of the 3D environment, where cell differentiation can be induced by the mechanical pressures and composition of the scaffold that mimics the natural interactions of the cells with the ECM [41,42,43,44,45]. It is known that the ECM plays a critical role in determining cellular phenotype not only through mechanical cues but also by the direct interaction of proteins with the cell surface receptors [41,42,43,44,46]. For example, human-derived Mesenchymal Stem Cells (hMSCs) have been shown to differentiate towards a neural lineage when grown in scaffolds of a stiffness of ~1 kPa [44].

One way to achieve this is by modulating the 3D matrix composition and stiffness that the cells are grown in to resemble the tissue of origin better. In this study, we looked at the neuro-differentiation effects of PEG-based hydrogels at 1.1 kPa on ADSCs for 14 days with the added adhesion motifs such as arginylglycylaspartic acid, the peptide trimer RGD found in collagen, laminin and fibronectin, which mediates the adhesion of many cells including neurons [47], and laminin-derived (YIGSR) peptide, which promotes neuronal cell binding [48].

## 2. Results

### 2.1. Cell Proliferation, Viability and Morphology

#### 2.1.1. ADSCs Proliferated in Both 2D and 3D Culture Conditions

**Figure 2:**
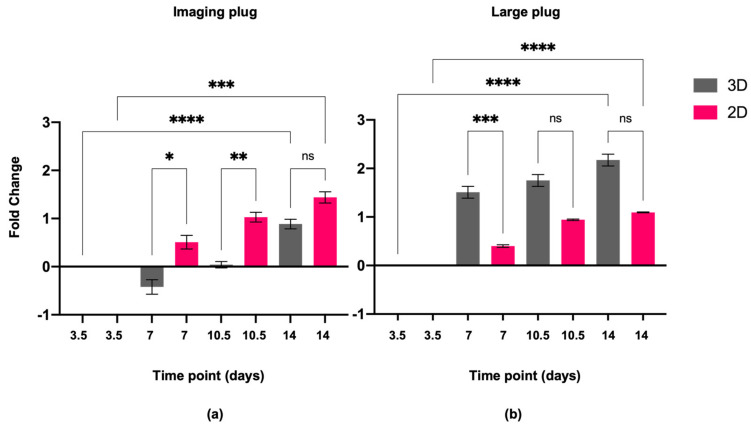
Fold change in cell activity over time relative to day 3.5 measured using alamar blue assay. Log2 scale has been used where the initial measurement obtained on day 3.5 equals zero, and the increase or decrease in measured parameters falls on the respective side of the *x*-axis. (**a**) Imaging plug; (**b**) large plug. Statistical significance defined as * = *p* ≤ 0.05, ** = *p* ≤ 0.01, *** = *p* ≤ 0.001 and **** = *p* ≤ 0.0001, ns: not significant.

Alamar blue viability assay showed that ADSCs remained viable and continued to proliferate in both 2D and 3D cultures for both the imaging plug (Figure 2a) and the large plug (Figure 2b), with 3D cultures showing higher proliferation rates in 3D than in 2D (Figure 2b). The imaging plug and large plug differences are further explained in the methods Section 4.1, but in brief, the imaging plug is the small version of the 3D construct, while the large plug is for proteome analysis. The concentration of cells was kept the same; however, the total number of cells differs depending on the size of the construct.

**Figure 3:**
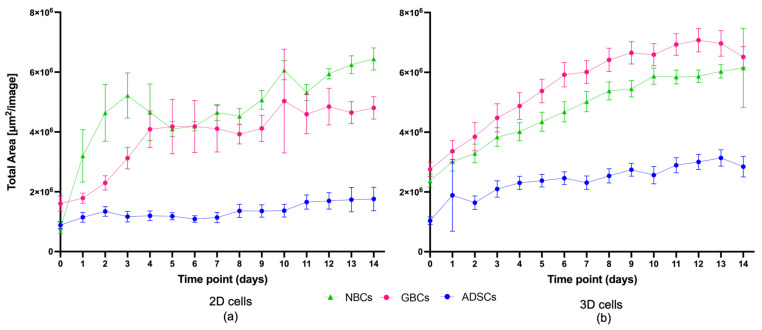

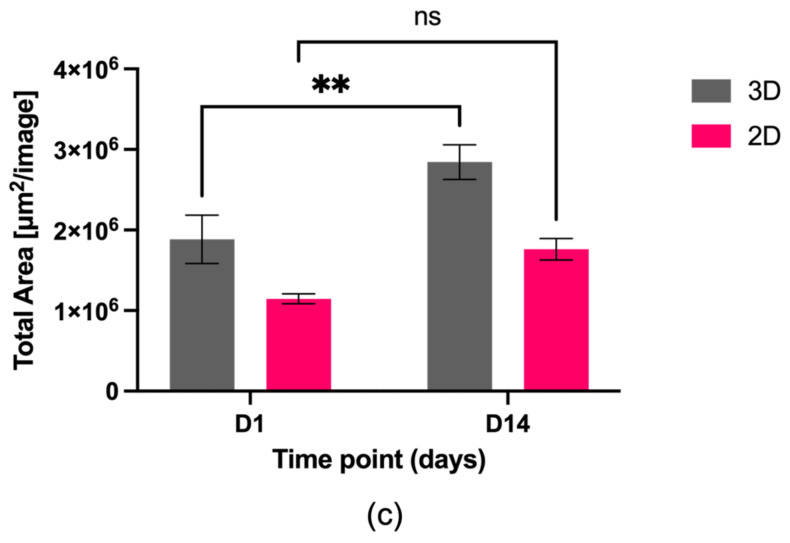
Graph showing the proliferation of cells over time as measured by total cell area for staining control cells GBC and NBC and for the ADSCs in (**a**) 2D and (**b**) 3D cultures (large plug). Error bars represent standard deviation. Green triangles—NBCs, pink circles—GBCs, and blue circles—ADSCs. (**c**) Graph showing the proliferation changes of ADSCs measured as total cell area comparing D1 and D14 when grown in 2D and 3D cultures (large plug). Statistical significance defined as ** = *p* ≤ 0.01, ns: not significant.

Live cell imaging analysis demonstrated that the staining control cells, U87MG glioblastoma cells (GBCs) and SHY5Y neuroblastoma cells (NBCs), proliferated over the 14-day period in both 2D (Figure 3a) and 3D (Figure 3b) conditions. There was a modest increase in ADSC numbers over 14 days in the 2D construct and a significant increase was seen in the 3D culture conditions, with a greater proliferation observed in the 3D construct at 14 days (Figure 3b,c).

Additionally, when looking at confluence data (Figure 3), an increase in total area covered by cells can be seen. 3D cells increased in area covered significantly over time between D1 and D14 in 3D (*p* ≤ 0.01) while the increase in 2D is not significant (Figure 3c).

#### 2.1.2. ADSCs Cell Morphology Changed in 3D Conditions

**Figure 4:**
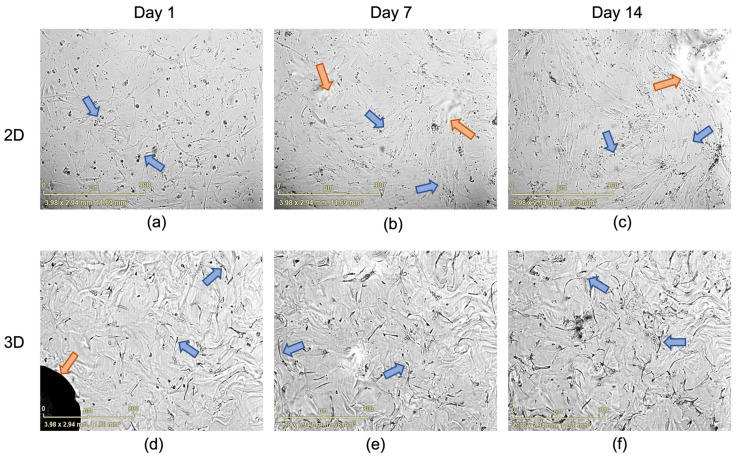

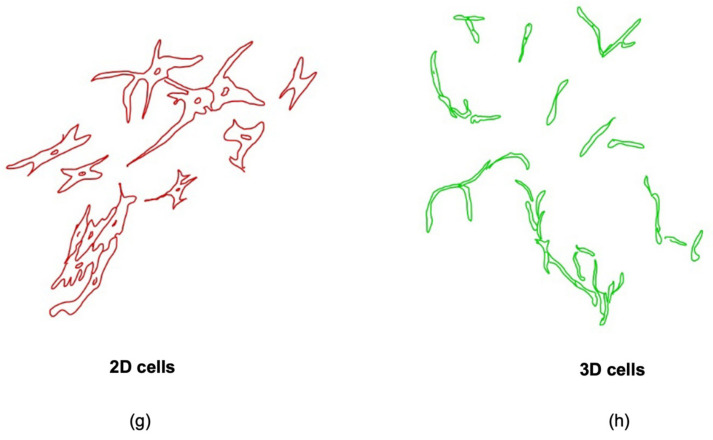
(**a**–**f**) Representative live cell images at time points D1, D7 and D14. Blue arrows indicate different cells and show differences between 2D and 3D; orange arrows show artefacts present in the images. The white blurry artefacts are glares and reflections, and the black round artefacts are bubbles; (**g**,**h**) Graphical representation of cell morphology changes between 2D and 3D cells. These were manually drawn by tracing the cell shapes on a digital tablet. Scale bar 800 μm.

Cell morphology is an important aspect to consider in cell culture, particularly when comparing a 2D growth environment to a 3D one. Clear morphological changes can be seen from Day 1 where ADSCs in 2D cell culture had an irregular fibroblastic-like structure, appearing as large, flattened cells with obvious centrally located nuclei (Figure 4a–c,g). In contrast, ADSCs grown in 3D constructs displayed changes in the membrane giving the cells a narrower appearance. The cells looked more spindly, with elongated profiles and cytoplasm less spread out, darker in colour, and overall taking up less area in the XY plane. Clear networks and branching out can also be seen between cells (Figure 4d–f,h). Additionally, in 2D, as the cells become more confluent, it becomes harder to distinguish morphologically individual cells, and instead, cells become a homogenous monolayer, while in 3D, the cells expand but retain their respective individual morphology.

### 2.2. Cell Characterisation

#### 2.2.1. Immunocytochemistry

**Figure 5:**
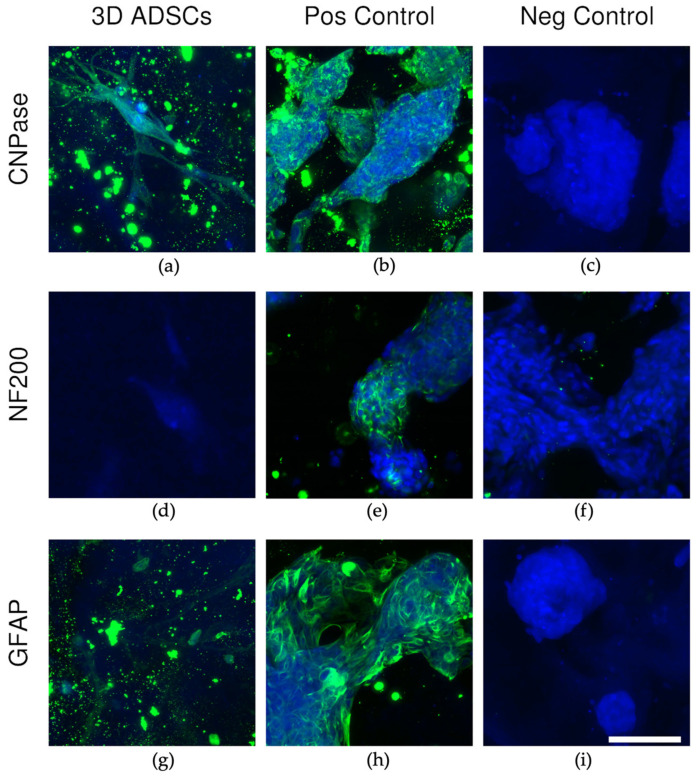

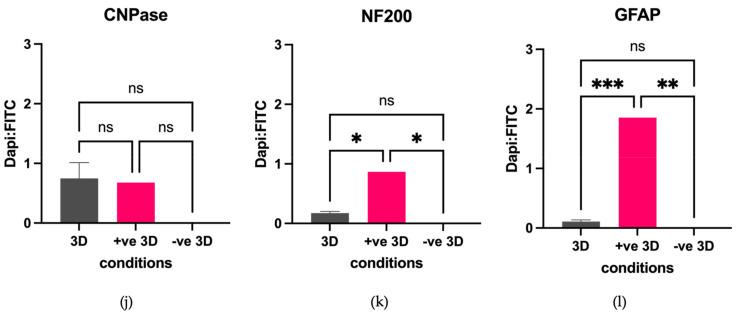
(**a**–**i**) Representative maximum intensity projection confocal microscopy images of immunocytochemistry staining of ADSCs in 3D with respective positive and negative staining controls for each antibody marker CNPase, NF200 and GFAP. Cells were imaged with a Nikon A1R inverted microscope using a S Plan Fluor LWD 20× 0.7NA objective. Fluorescence was captured with a laser at 405 nm excitation and PMT detector (425–475 nm) for DAPI (blue), and 488 nm excitation and GaAsP detector (500–550 nm) for AlexaFluor488-conjugated secondary antibodies (green). Scale bar = 100 μm. Please note that control cells are smaller than ADSCs; (**j**–**l**) immunocytochemistry marker expression for 3D cells. Marker expression was measured from sum intensity projections of wide-field fluorescence images of the whole 3D plug and is displayed as the fraction of the percentage area of FITC (AlexaFluor488-conugated secondary antibody) over the percentage area of DAPI-labelled nuclei. No statistical significance *p* > 0.05; statistical significance *, *p* ≤ 0.05; statistical significance **, *p* ≤ 0.01; statistical significance ***, *p* ≤ 0.001; statistical significance, ns: not significant.

Immunocytochemistry imaging (Figure 5) showed expression of the CNPase marker only in the 3D-grown ADSCs. All other markers remained negative.

#### 2.2.2. Proteomics

Proteomic analysis using MaxQuant and LFQ Analyst revealed clear proteome changes between ADSCs grown in 2D vs. 3D environments.

**Figure 6:**
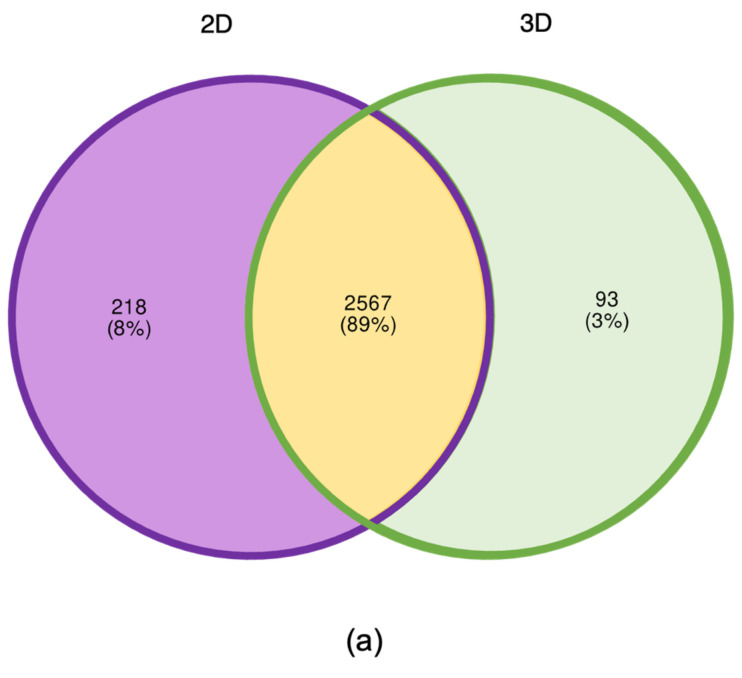

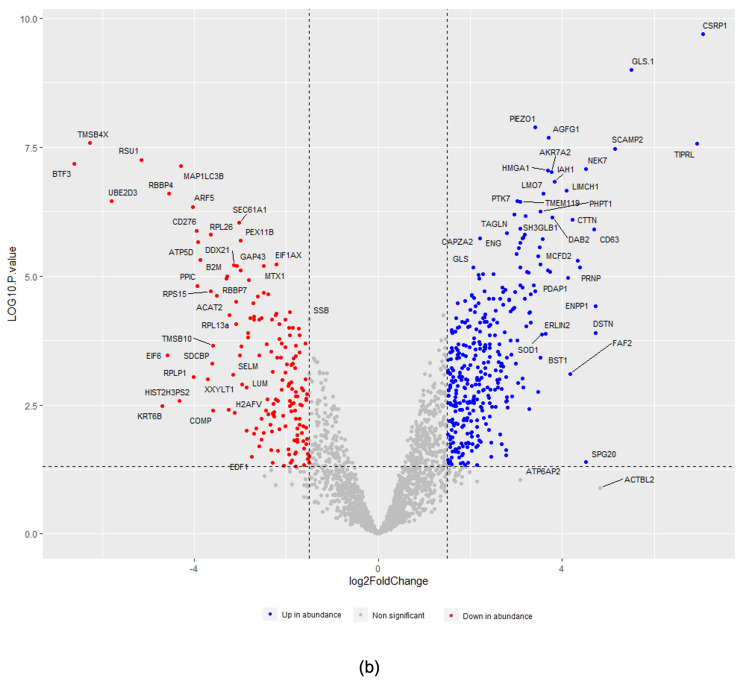
(**a**) Venn diagram showing total number of proteins detected in both 2D and 3D sample groups as well as the total number of proteins that were detectable in 2D sample group and were not in the 3D sample group and all proteins that were detectable in 3D sample group that were not detected in the 2D sample group; (**b**) Volcano plot showing total number of proteins detected with increase or decrease in abundance in 3D samples compared to 2D samples. Blue dots represent proteins that have increased in abundance by >1.5 fold with a *p*-value of <0.05 in 3D samples compared to 2D samples; red dots proteins that have decreased in abundance > 1.5 fold with a *p*-value of <0.05 in 3D samples compared to 2D samples. Gray are all non-significant detected proteins.

A total of 2878 proteins were identified, with 218 proteins only present in 2D samples and absent in 3D samples, and 93 present in 3D samples but absent in 2D samples (Figure 6a). Additionally, of the total 2878 proteins identified, 2291 proteins changed in abundance with 439 proteins changed in abundance by >1.5-fold with *p*-value < 0.05 meaning 15% of all proteins detected significantly changed in abundance by at least 1.5-fold (Figure 6b).

Supplementary S1 (Table S1) includes all 439 proteins that changed in abundance with respective log2 fold changes and *p*-values.

**Figure 7:**
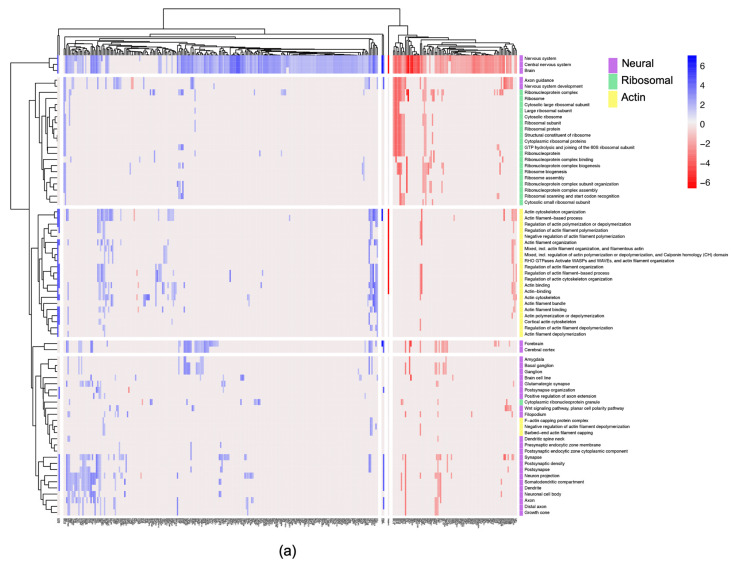

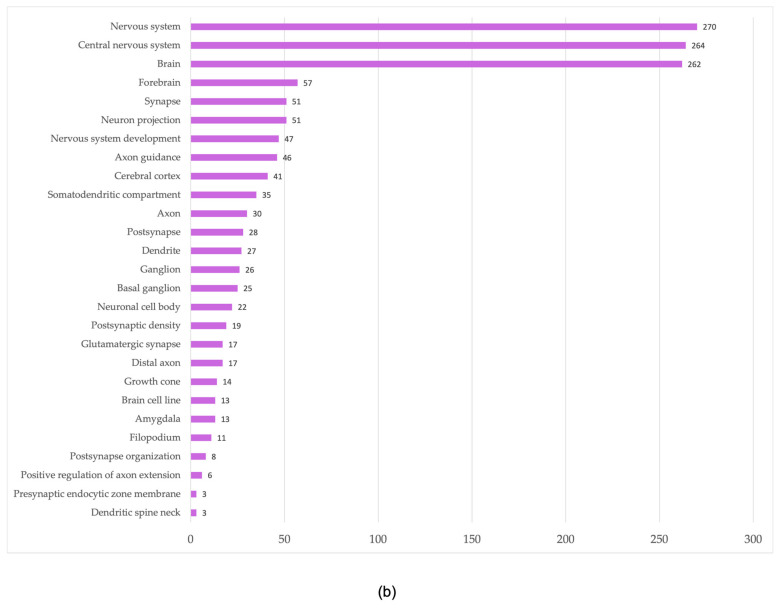
(**a**) Heatmap showing all proteins involved in neural, actin and ribosomal processes with respective fold change. Hierarchical clustering and further division into similarity groups was conducted. Blue indicates increase in abundance, red decrease in abundance, purple neural functions, green ribosomal functions and yellow actin functions. (**b**) Number of proteins identified with roles in neural functions revealed during functional and network analysis.

While functional enrichment analysis of all 439 significant proteins using StringDB revealed quite broad changes, changes in actin, ribosomal and neural-related functions are notable (Supplementary S2). The heatmap displayed in Figure 7a shows the different functional enrichments of the proteins detected relating to actin, ribosome and neural processes and their respective log2 fold change, and Figure 7b shows in more detail the number of proteins detected involved in each function.

A total of 67 proteins with changed abundance are related to actin; of these 67, 45 are related to both actin and neural processes, of which 36 proteins increased in abundance, and 9 decreased in abundance (Supplementary S2).

Supplementary S2 includes a table with all the proteins and functions included in Figure 7 heatmap and graph (Table S2), the permalink to the StringDB network analysis results and an additional heatmap of all functional enrichment processes detected using StringDB with log2 fold change in all proteins (Figure S1).

## 3. Discussion

The aim of this study was to examine ADSCs’ cellular and molecular differences when grown in 2D or 3D environments where the 3D environment is designed to mimic the density and elasticity of brain tissue. It was hypothesised that there would be biological differences at the cellular level in growth patterns and morphology as well as complementary changes in the analysed proteome. The data confirmed the hypothesis that the 3D environment did indeed have a significant effect on the ADSCs morphology and protein abundances when compared to 2D-grown ADSCs.

Cell viability was unchanged between the 2D and the 3D gels, and cells exhibited increased proliferation as shown by an increase in alamar blue fluorescence and by an increase in the area covered by cells in the 3D-grown cells (Figure 2 and Figure 3) compared to the cells in the 2D environment. Furthermore, morphological changes in the cells were clearly seen in the 3D environment compared to the ones in 2D. In the 3D environment, cells became spindle-like and aligned with one another, showing elongation, branching out and a low nuclei-to-cytoplasm ratio, which are key morphological characteristics of neural cells, implying that these cells are differentiating down a neural lineage by just being in a 3D environment.

Immunocytochemistry analysis showed that ADSCs started to express CNPase, a well-known oligodendrocyte marker, in the 3D environment. Structural and neural changes were further confirmed by the proteomics analysis, which showed significant proteome changes between cells grown in 2D and 3D environments. There were over 2870 proteins identified and 439 significantly changing in abundance, with the most striking changes were observed in proteins annotated as being involved in neural, actin and ribosomal processes.

### 3.1. ADSCs in 3D Matrices Show Increased Cell Viability and Morphological Changes Indicative of Neural Differentiation

It has been previously reported that neural precursor cells grown in PEG-based matrices have a higher metabolic activity, lower apoptotic activity and higher cell proliferation rates [49], as well as enhancing neural stem cell (NSCs) survival, proliferation and differentiation compared to those grown in 2D environments [50]. The alamar blue assay, a widely used viability and proliferation test, showed that the cells remained viable and continued to proliferate over the 14-day period in our study, with the cells grown in 3D conditions having higher proliferation rates than those in 2D conditions (Figure 2). Additionally, cell area coverage analysis showed an increase in coverage over time, with 3D cells having a significantly increased cell coverage between day 1 and day 14 (Figure 3), showing that the cells are thriving in the 3D environment.

It is also widely known that the cell environment plays a key role in many cell functions and influences proliferation, differentiation, migration and morphology [51]. When cells are grown in 2D conditions, they are forced flat onto a hard surface where they are only attached to the XY plane and have no interactions or pressures from the Z plane; therefore, they do not have anything “on top of them” and are forced to extend their cytoplasm in order to have more attachment points [52]. On the other hand, when cells are grown inside a matrix in a 3D conformation, cells start to interact with the matrix in an XYZ axis, having cues all around them. In 3D conditions, the cells are suspended within the matrix, where they can move and migrate and interact with other cells, rather than being forced to attach to a 2D surface that cannot be modified by the cells [52]. Furthermore, the stiffness of polystyrene or glass surfaces used to grow monolayer cultures is multiple orders of magnitude greater than any soft tissues found in the human body. This environment provides atypical stimuli that affect cellular development [53]. This is especially important in the context of neural development, as brain tissue represents one of the softest tissues in the body [54].

3D matrices are known to mimic better in vivo conditions, and cells are known to adopt morphologies more resemblant to those occurring in the body as well as responding to the mechanical cues of the matrix [36]. The ADSCs grown in the 3D matrices have also shown notable morphological changes, with cells going from large, flat, “fried-egg” like shapes with centrally located nuclei and large cytoplasm-to-nuclei ratio to thin, elongated, spindle-shaped cells with low cytoplasm-to-nuclei ratio and branching out creating networks between cells (Figure 4), which are morphological features usually seen in neural cells. In addition, mechanical cues play an important role in stem cell differentiation. It has been previously shown that substrate stiffness can direct attachment, survival, growth and differentiation of MSCs [44]. For example, MSCs can undergo osteogenesis when placed in stiffer substrates [55,56,57] and differentiate towards neural lineage when placed in softer matrices [41,44], with MSCs going towards neuronal lineage in ~1 kPa stiffness matrix and towards glial lineage when in ~10 kPa matrices [44]. These results suggest that these cells may be differentiating down a neural lineage by the PEG-based matrices.

### 3.2. Immunocytochemistry and Proteome Changes of ADSCs in 3D Matrices Are Indicative of Neural Differentiation

CNPase is a myelin-associated protein that is expressed in pre-oligodendrocytes and oligodendrocytes and is widely used as a marker for early oligodendrocyte differentiation and myelin formation [58,59,60]. Furthermore, CNPase has been previously detected in ADSCs undergoing chemical differentiation in 2D environments [61]. In the current study, CNPase was detected in the immunocytochemistry results for the ADSCs grown in 3D only (Figure 5), and it was found to be slightly increased (0.9 log2 fold change) in the proteome findings in the 3D-grown cells suggesting that perhaps the cells are starting to differentiate towards oligodendrocytes or that there may be myelin formation occurring. GFAP and NF-200 immunocytochemistry did not show increases in the ADSCs grown in 3D. However, it should be noted that these are both mature structural cell markers, and the ADSCs may not be at that stage of differentiation yet, given how long neural cells take to differentiate, with cortical neurogenesis taking around 108 embryonic days to complete [62].

### 3.3. Structural Proteins Expression Involved in Neural Differentiation

The proteome analysis showed protein abundance changes related to neural, actin and ribosomal functions. Interestingly, network analysis detected over 250 proteins with statistically significant changes in abundance relating to the nervous system and nervous system processes (Figure 7), of which 45 proteins are also involved in actin-related functions (Supplementary S2 Table S2). Actin is an essential component of cell cytoskeleton; it has an important role in cell survival, morphology and movement. Actin filaments, in conjunction with other proteins, provide mechanical support, assist in sensing environmental cues and tracking the movement of intracellular materials, such as internalised membrane vesicles, and assist in cell migration and division [63]. More specifically, in the neural context, microtubules, neurofilaments and F-actin are the main filaments of the neuronal cytoskeleton. Actin, in particular, is involved in neuronal outgrowth, morphology and synaptic function, playing a key role in establishing and maintaining neuronal polarity with many actin-regulating proteins influencing neuronal morphology and plasticity [64,65]. Neurons’ polarised morphology is instrumental to their ability to process and transfer information between dendrites and axons. These are long and highly branched structures extending from the neuronal cell’s body and reaching up to hundreds of microns in length, forming a widespread and complex arbour [64,66,67]. Additionally, the growth cones within neurons detect and interpret extracellular signals that guide the growth and elongation of neurons [68]. These findings, supported by the observed neural-like morphological changes in the ADSCs grown in 3D PEG-based matrices (Figure 4), suggest that the cells are going through structural and shape changes indicative of neural differentiation.

Of particular interest is CSRP1 protein, also known as CSP1, it was the protein most increased in abundance detected in the whole dataset. It increased by 7.3 log2 fold change (Figure 6b) in 3D-grown cells. CSRP1 has diverse roles in cellular development, from suppressing cell proliferation, protecting cells from stress-induced death, regulating cell movement [69,70] and playing a role in actin dynamics by interacting with actin to regulate actin filament bundling [71,72]. In the nervous system context, CRP1 is the only protein of the CRP protein family to be found in the CNS [73]. It colocalizes with actin in growth cone filopodia in neurons playing a role in its formation; increased CRP1 expression has been found to increase filopodia formation and dendritic growth in neurons, and its absence has been found to cause the opposite, with the deletion of CSRP1 gene causing inhibition of filopodia formation and dendritic growth in neurons [73].

Another protein group of interest is the ADF/Cofilin proteins that are well-known regulators of actin dynamics and are highly expressed in growth cones [74,75]. They are involved in growth cone motility, axon growth and neurite extension during early neural development [76,77,78,79]. ADF/Cofilin proteins have also been recently recognised as promising target proteins to regenerate axons in the adult nervous system, given their critical role in F-actin binding, severing and depolymerising activities during early neuronal development [80]. Furthermore, cofilin knockdown models resulted in neuron polarity defects [75] and ADF/Cofilin are known to regulate synaptic function through their effect on dendritic spines [81]. Interestingly, our results showed that ADF (labelled as DSTN) was one of our top six proteins increased in abundance in the 3D-grown cells with a log2 fold change of 4.72. Cofilin (labelled as CFL1) was also found to be increased by 2.02 log2 fold.

Another interesting finding is the high increase in the actin-binding protein Cortactin (labelled as CTTN). In our dataset, Cortactin was increased by a log2 fold change of 4.23, making it one of the top 12 most increased proteins in the dataset. CTTN is an actin-binding protein that regulates actin cytoskeletal networks and is essential for endocytosis, cell migration, adhesion, synaptic organisation and cell morphogenesis [82]. It is found in the dendritic spines [83] and more specifically plays an important role in pre- and postsynaptic structures and in neuron-specific functions like axon guidance, synaptogenesis and growth cone formation as well as in functional and structural synaptic plasticity [84,85,86,87,88]. CTTN loss is also associated with a reduction in dendritic spine numbers [83], and it is enriched in both axonal and dendritic growth cones of young neurons [89]. Studies have also found that CTTN is enriched in the central region of all neurite growth cones prior to neurons developing into axons [90].

Several other actin-binding proteins, which are involved in similar pathways, are also increased in abundance in this proteomic data, which further supports our findings. CAP2 increased by 3.16 log2 fold in 3D-grown cells, and it is expressed in growth cones, dendrites and postsynaptic terminals. It is also involved in dendrite morphology regulation, spine development and synaptic plasticity in neurons [91,92,93]. ADD1, increased by 2.65 log2 fold, is an actin-binding protein of the subcortical neuronal cytoskeleton and plays a role in axonal diameter maintenance [94] and synaptic plasticity [95]. DBN1, also known as drebrin, was increased by 1.59 log2 fold in the 3D-grown cells and has a role in neuron growth and brain development. It is present in the dendritic spines of excitatory synapses, and it is mainly found during early brain development in the dendritic spines of immature neurons [96,97,98].

Lastly, β3-Tubulin (TUBB3) was found to have increased by 1.51 log2 fold in the 3D-treated cells. TUBB3 is a major component of the neuronal cytoskeleton, and it is highly expressed in microtubule during neural development, playing a critical role in maintenance, maturation and proper axon guidance, and it has been long used as a neuronal marker in [99,100,101,102,103].

These proteome changes, together with the observed morphological changes in Figure 4, suggest that the cells’ cytoskeleton is significantly rearranging in response to its environment, and the cells may be differentiating towards neural cells forming axons and dendrites.

### 3.4. Ribosomal Protein Involvement in Neural Differentiation

The proteomics findings also clearly indicate considerable changes in the abundance of proteins involved in ribosome and translational processes (Figure 7a). Ribosome biogenesis and regulation of protein synthesis are known to be of major importance when it comes to the modulation of cell behaviour [104]. It is well known that stem cells possess the ability to self-renew and differentiate. However, the self-renewal potential diminishes once the cells start differentiating towards a more specific lineage. The same applies to proliferative capacity. The balance between molecular processes responsible for maintaining pluripotency and directing cell fate rapidly shifts to accommodate these changes [105]. The most striking decrease in abundance out of all proteins in our dataset can be observed in the basic transcription factor 3 (referred to as BTF3). It sustained a −6.61 log2 fold change in proteome of cells grown in 3D environment. BTF3 has been found to be responsible for maintaining stem-like characteristics, and its lowered presence has been linked to loss of self-renewal capacity in differentiating stem cells [106,107]. Additionally, several proteins associated with formation of cytoplasmic ribonucleoprotein granule have been shown to increase in abundance in the 3D environment. One of them was the far upstream element-binding protein 2 (here referred to as KHSRP), which increased by 3.09 log2 fold. It is a KH-type splicing regulatory protein, responsible for RNA binding and the resulting decay of mRNAs with AU-rich elements found in the 3′ untranslated region (UTR) [108]. KHRSP has been shown to be highly expressed in the brain tissue. It plays an important role in neuronal development by regulating axonal branching and elongation, while its deficits were linked to impaired neuronal development [109,110].

Proteome analysis also demonstrated decrease in abundance of several proteins that are known to be components of small and large ribosomal subunits. This indicates that the ribosome biogenesis levels are not what is expected of fully differentiated cells. The 14-day incubation period, without neural induction media or supplements, was not sufficient for the ADSCs to become entirely committed. This was confirmed using the viability (Figure 2) and proliferation (Figure 3) assessments, where slow increase in the parameters are still visible at the time of final measurements for both 2D and 3D cells. If the cells were at a further stage in their differentiation, higher abundance of ribosomal proteins and higher translational efficiency would be expected [105]. Interaction analysis, performed using StringDB, revealed strong associations between the ribosomal subunit proteins and the before-mentioned BTF3 protein. Therefore, the decrease in ribosome biogenesis can be linked to the reduction in BTF3 synthesis, which subsequently can result in lower global biogenesis levels. At the same time, some proteins from the group of eukaryotic translation initiation factors (eIFs), such as EIF4H, EIF3E, EIF3F, and EIF3M, have increased significantly in abundance with log2 fold changes ranging from 1.73 to 3.32 depending on the factor. Translation is highly coordinated by the assembly of eIFs at the 5′ end of mRNAs. The EIF-3 complex has been shown to play a crucial role in regulation of mRNA translation by controlling various steps of protein synthesis including initiation, elongation and termination [111]. The function of EIF4H in protein synthesis is executed by enhancing helicase activity, which facilitates mRNA recruitment process in the ribosome [112]. Increase in abundance of proteins responsible for initiating translation shows that even after the short 14-day incubation time, some alterations in the cellular mechanisms of ADSCs are present. As much as the exact direction of these changes cannot be established with certainty, the 3D environment is most definitely making an impact on the cells.

In summary, this study has shown that ADSCs grown in a PEG-based matrix mimicking brain stiffness underwent significant cytoskeletal changes. Cells started to rearrange, and the formation of dendrites and axons is suspected, given the morphological and proteome changes. Furthermore, early oligodendrocyte marker expression also suggests that the cells are starting to differentiate towards the oligodendrocyte lineage. It is possible that the cells are starting to differentiate into multiple populations of cells rather than just one type of cell, given the multicellular nature of the brain. These findings are promising and set a precedent to explore this area further. The addition of chemical differentiation mixtures as well as longer time periods would be the logical next steps.

## 4. Materials and Methods

ADSCs from a single donor were isolated and expanded as previously described [113] with approval from the UTS Human Research Ethics Committee (Ethics number 2013000437). Written informed consent was acquired for donor lipoaspirate release for research purposes only. After isolation, and prior to experiments, the cells were maintained in DMEM/F12+Glutamax media (Gibco, Life Technologies, Carlsbad, CA, USA) with 10% heat inactivated FBS (Gibco, Life Technologies, Carlsbad, CA, USA) and incubated at 37 °C at 5% CO_2_. Cells used for these experiments were between passage ten and twelve.

At passage 10–12, cells were lifted from the tissue culture flasks using TrypLE express (12604 Gibco, Life Technologies, Roskilde, Denmark) and either re-plated into 96-well plates (2D) or prepared for bioprinting (3D) following manufacturer’s instructions.

Once the cells were re-plated in 2D or bioprinted, they were maintained in similar conditions as above with the addition of 1% antibiotics/antimycotics (ABAM, Gibco life technologies, Carlsbad, CA, USA) to the media; media was changed every 84 h using the same maintenance media and incubated at 37 °C at 5% CO_2_.

### 4.1. 3D Bioprinting of ADSCs in PEG-Based Hydrogels

ADSCs were 3D printed in a PEG-based hydrogel using a RASTRUM bioprinter (Inventia, Sydney, Australia). The cell plugs were printed into 96-well plates following the manufacturer’s instructions.

In brief, a large plug and imaging plug were printed at ~1.1 kPa containing RGD and YIGSR peptides (matrix code PX02.21P). RGD and YIGSR were included in the system given that the peptide trimer RGD is found in collagen, laminin and fibronectin, which mediates the adhesion of many cells including neurons [47], and laminin-derived (YIGSR) peptide is known to promote neuronal cell binding [48].

**Figure 8:**
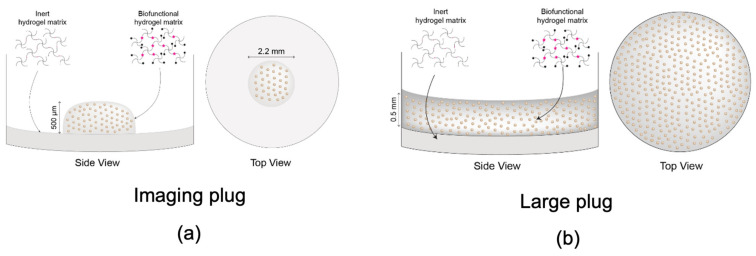
Visual representation and dimensions of the different constructs provided by Rastrum. (**a**) Smaller sized plug, referred to as imaging plug used for immunocytochemistry; (**b**) Larger size plug, referred to as large plug (used for viability assays, live cell imaging and proteomics). Diagrams adapted with permission from Inventia Life Science.

Cells were seeded at a concentration of 10 million/mL. The imaging plug consisted of a small volume of hydrogel with embedded cells in the centre of the well measuring 0.5 mm in height and 2.2 mm in diameter (Figure 8a). The large plug occupied the well completely, measuring 0.5 mm in height and 5 mm in diameter (Figure 8b). Negative controls were included as 2D-seeded cells. Cells were lifted from the tissue culture flasks using TrypLE express (12604 Gibco, Life Technologies, Roskilde, Denmark) and re-plated into 96-well plates in 2D conditions at 10 million/mL, the same concentration as 3D cells. These were grown in parallel and treated the same way. The only difference was the 3D construct vs. 2D environment.

Positive controls for immunocytochemistry were included and printed in parallel to the ADSCs following the same method. These are further explained in the immunocytochemistry Section 4.4.1.

### 4.2. Cell Morphology: Incucyte Imaging

Live images of the same Z plane were taken daily using the organoid program in the Incucyte^®^ S3 Live-Cell Analysis Instrument for 14 days, and morphological changes were visually assessed.

Cell confluence was assessed as the area covered by cells in each image (μm^2^/image). This was conducted using the instrument inbuilt analysis software. The parameters used to assess ADSCs confluence were the following: Radius 200; Sensitivity 70; Edge sensitivity 0; Hole fill (μm^2^) 500; Adjust size (pixels) 0. After the initial analysis was finalised by the instrument, all images in all time points were manually checked for artifacts that would not accurately represent the confluence. Common artifacts found in the images were glare (Figure 4C) and bubbles (Figure 4D), which prevented the camera from taking an accurate photo of the cell coverage. Further analysis and graphing were performed using the data exported from the Incucyte^®^ proprietary software version 2022B Rev1. Averages of total area for wells of each cell type and per time point, with associated standard deviations for both for 2D and 3D models, were plotted. The dataset was assessed for normality using Shapiro–Wilk test and statistical significance was subsequently determined using parametric two-way ANOVA with Tukey’s multiple comparisons. GraphPad PRISM software version 9.5.0 was used for data visualisation (Figure 4).

### 4.3. Cell Viability and Proliferation: Alamar Blue

Cell viability assay was performed at four different time points: D3.5, D7, D10.5 and D14 using an Alamar blue assay. Alamar blue is a non-toxic cell viability assay that detects metabolically active cells. When Alamar blue is added to cells, if cells are metabolically active, the main active ingredient resazurin is reduced to resorufin, and the solution becomes red in colour and highly fluorescent.

Alamar blue (10% in media) was added to the cells and left to incubate for 16 h to allow enough time to penetrate through the 3D matrices. To keep variables to a minimum, the same was performed on the 2D cells. Negative control wells were included; these only contained the alamar blue and media mixture. After the incubation period, the alamar blue and media mixture was transferred to a different 96-well plate to keep cellular growth environment as undisturbed as possible from outside factors. The collected alamar blue media was then measured using the fluorescence bottom-up mode in a Tecan M200 Plate Reader using 530–560 nm excitation and 590 nm emission wavelengths. The results were averaged across the 96-wells, and data was normalised to the negative controls. The data was analysed as fold change ratio values from D3.5 to standardize and allow for comparison. The dataset was assessed for normality using Shapiro–Wilk test and, due to the assumption not being met, non-parametric Kruskal–Wallis test was performed to determine significance. GraphPad PRISM software version 9.5.0 was used for data visualisation (Figure 2).

### 4.4. Cell Characterization

#### 4.4.1. Immunocytochemistry

Cells from the imaging plug were fixed using 10% formalin for 30 min prior to washing and storing in PBST + 0.1%*w*/*v* sodium azide at 4 °C.

For staining, cells were first placed in PBST (0.01M PBS and 0.1% Triton X-100 (BDH #30632) at pH 7.4) for 1 h at room temperature. Primary antibodies were diluted in PBG (0.1M PBS, pH 7.4, 0.1% Triton-X, 2% NGS, 1% BSA (Sigma-Aldrich, St. Louis, MO, USA, #A9647)) and were added to the relevant wells and incubated at 4 °C for 3 days. Primary antibodies were rabbit anti-glial fibrillary acidic protein (GFAP) (1/500, Dako, Glostrup, Denmark #Z0334) as an astrocyte marker; mouse anti-2′,3′ cyclic-nucleotide 3′ phosphodiesterase (CNPase), (1/100 Abcam, Cambridge, UK, #ab6319-100) as an oligodendrocyte marker; and mouse anti-Neurofilament 200 (1/50, biosensis CM998100) as a mature neuron marker.

After primary antibody incubation was completed, cells were then washed with three changes of PBST for 30 min and incubated with goat anti-mouse AF488 (1/200, Invitrogen, Carlsbad, CA, USA #A11001) or goat anti-rabbit AF488 (1/ 200, Invitrogen, Carlsbad, CA, USA #A11008) secondary antibodies in PBG for another 3 days at 4 °C. Following an additional two 20 min washes with PBST, cells were incubated with Hoechst (1/5000 Invitrogen, Carlsbad, CA, USA) for 30 min to stain the nuclei and finally washed three times with PBST for another 30 min each and stored in antifade/glycerol at 4 °C until imaged.

Positive staining control cells at 10 million/mL conc were included in all staining runs. Glioblastoma U87MG cells were used for GFAP- and CNPase-positive staining controls. Neuroblastoma SHSY-5Y cells were used for NF200-positive staining controls. Both U87MG and SHSY-5Y cells were grown in separate plates to the experimental cells; however, the cells were grown and stained in parallel with the experimental plates for each antibody and were fixed and stained following the same protocol as the experimental cells. In both as 2D and 3D environment, U87MG and SHSY-5Y cells were grown in a 96-well plate with DMEM/F12+Glutamax media (Gibco, Waltham, MA, USA) enriched with 10% heat-inactivated FBS (Sigma-Aldrich, St. Louis, MO, USA) until confluent.

Brightfield and wide-field fluorescence microscopy was performed using a Nikon Ti inverted microscope with a 10× 0.3 numerical aperture Plan Fluor objective, NIS Elements acquisition software (version 5.30.06) with a solid state Lumencor illumination source and a Nikon DS-Qi2 CMOS camera. Six 1024 × 1024 field of views were captured covering the area of each imaging plug and stitched using the NIS Elements acquisition software with default overlap settings. Series of images were captured through the *z* dimension using a step size of 5.6 µm.

Wide-field fluorescence images were processed using Clarify.ai and Denoise.ai algorithms using the NIS Elements acquisition software. FIJI (FIJI is just ImageJ) version 1.53t [114] was used for image analysis. Where appropriate, z-stacks were corrected for axial drift using the Linear Registration with SIFT plugin with an expected translation transformation. Sum intensity projections for FITC and DAPI channels were thresholded using the default algorithm to create a binary mask and area fraction was measured. For immunolabelled images (FITC channel), binary masks were processed using Smooth and Fill Holes before area fraction was measured. To eliminate non-specific secondary antibody aggregates from measurements, only particles with a pixel size of larger than 100 pixels^2^ and a circularity of 0–0.8 were quantified. Marker expression was measured from sum intensity projections of wide-field fluorescence images of the whole 3D plug and is displayed as the fraction of percentage area of FITC (AlexaFluor488-conugated secondary antibody) over the percentage area of DAPI-labelled nuclei. One-way ANOVA with multiple comparison was conducted using Bonferroni’s multiple comparison test. No statistical significance *p* > 0.05; statistical significance *, *p* ≤ 0.05; statistical significance **, *p* ≤ 0.01; statistical significance ***, *p* ≤ 0.001; statistical significance ****, *p* ≤ 0.0001.

Representative images were captured using a Nikon A1R inverted confocal microscope with a 20× 0.7 numerical aperture LWD S Plan Fluor objective and NIS Elements acquisition software. DAPI was imaged with an excitation of 405 nm and emission detected with a PMT detector at 425–475 nm. AF488 was imaged with an excitation of 488 nm and emission detected with a GaAsP detector at 500–550 nm. To account for differences in labelling intensity in these qualitative images, each expression marker was imaged using the following settings: CNPase samples were captured with a 488 nm laser intensity of 4.16, gain of 44 and offset of −3 and a 405 nm laser intensity of 18.95, gain of 150 and offset of −5; NF200 samples were captured with a 488 nm laser intensity of 6.71, gain of 44 and offset of −3 and a 405 nm laser intensity of 13.22, gain of 150 and offset of −2; GFAP samples were captured with a 488 nm laser intensity of 1.75, gain of 39 and offset of −3, and a 405 nm laser intensity of 18.95, gain of 150 and offset of −2. Z-stacks were acquired with a step size of 3 µm. Fluorescence images were processed using the Denoise.ai algorithm using the NIS Elements acquisition software, and Maximum Intensity Projections were created. In Figure 5, the displayed dynamic range for FITC for 3D samples is 0–550, whilst for positive controls the displayed dynamic range for FITC is 0–1000.

#### 4.4.2. Proteomics

##### Protein Extraction

Cells were released from the 3D large plugs using Rastrum cell retrieval protocol provided by the company. In brief, media from printed 3D cell models was discarded, cells were washed with PBS and cell retrieval solution was added to the wells. The wells were then incubated at 37 °C for 30 min. Cells were then collected by pipetting up and down in each well and then were transferred to the collection tubes. The wells were then further washed with PBS and remaining cells were combined in the tubes. Cells were then centrifuged, and supernatant was removed. The cell pellets were then frozen until ready to be used for proteomics. Six wells were pooled to make one proteomics sample.

Once ready, samples were defrosted and resuspended in 1% SDC, 5 mM TCEP, 10 mM IAA, 100 mM HEPES pH 8.5, heated to 95 °C for 5 min and incubated for an hour at room temperature. After the incubation, 0.1 μg of trypsin was added to 10 ug of sample and incubated at 37 °C overnight. The peptides were then recovered using SDB-RPS-based stage tip column method, which is a modified protocol from Rappsilber et al., 2007. The digested cell were centrifuged at maximum speed for 5 min to digest any insoluble material, and 10× the volume of digest was the volume of SPE load buffer added (90% acetonitrile, 1% trifluoroacetic acid). The sample was mixed by pipetting up and down and was then added to the stage tip column that contained one disc of SDB-RPS cut with an 18-gauge blunt end needle. The liquid was centrifuged through the disc at 5000 rpm until all liquid moved through. Following this, two washing steps were performed to help wash any contaminants and salts from the column and bound peptides. Firstly, 100 μL of SPE load buffer were passed through at 5000 rpm until all liquid moved through followed by second wash using 100 μL of SPE wash buffer (10% acetonitrile, 0.1% trifluoroacetic acid). After, the peptides were eluted directly into the injection vials by washing the column with 50 μL of SPE elution buffer (71 μL of 1M ammonia solution, 800 μL of 100% acetonitrile, 129 μL of water) and centrifuging at 5000 rpm until all liquid passed through the column into the vials. The vials containing the peptides were then placed into the vacuum centrifuge (Savant DNA 120, SpeedVac Concentrator, Thermo Scientific, Carlsbad, CA, USA) to evaporate all liquid. Once samples were dry, the peptides were resuspended using 25 μL of MS loading solvent (2% acetonitrile, 0.2% trifluoroacetic Acid) and samples were ready to be analysed using LC-MS/MS.

##### LC-MS/MS Analysis

Using an Acquity M-class nanoLC system (Waters, Milford, MA, USA), 5 µL of the sample was loaded at 15 μL/min for 3 min onto a nanoEase Symmetry C18 trapping column (180 μm × 20 mm) before being washed onto a PicoFrit column (75 μm ID × 350 mm; New Objective, Woburn, MA, USA) packed with SP-120-1.7-ODS-BIO resin (1.7 μm, Osaka Soda Co., Tokyo, Japan) heated to 45 °C at 300 nL/min. Peptides were eluted from the column and into the source of a Q Exactive Plus mass spectrometer (Thermo Scientific, Carlsbad, CA, USA) using the following program: 5–30% MS buffer B (98% acetonitrile + 0.2% formic acid) over 90 min, 30–80% MS buffer B over 3 min, 80% MS buffer B for 2 min, 80–5% for 3 min. The eluting peptides were ionised at 2400 V. A data-dependant MS/MS (dd-MS2) experiment was performed, with a survey scan of 350–1500 Da performed at 70,000 resolution for peptides of charge state 2+ or higher with an AGC target of 3 × 10^6^ and maximum injection time of 50 ms. The top 12 peptides were selected fragmented in the HCD cell using an isolation window of 1.4 *m*/*z*, an AGC target of 1 × 10^5^ and maximum injection time of 100 ms. Fragments were scanned in the Orbitrap analyser at 17,500 resolution, and the product ion fragment were masses measured over a mass range of 120–2000 Da. The mass of the precursor peptide was then excluded for 30 s.

##### Data Processing and Analysis

The MS/MS data files were searched using MaxQuant (version 2.0.3.0) hosted on the Galaxy Australia platform against the UniProt *Homo sapiens* database (downloaded on 23 March 2023) using the following specific parameters settings. Min. peptide length: 7; Max. peptide mass (Da): 4600; Min. unique peptides: 0; Calculate peak properties: false; Match between runs: True; Match time window (min): 0.7; Match Ion Mobility Window: 0.05; Alignment time window (min): 20; Alignment ion mobility: 1; Match unidentified features: False; Include contaminants: True; Decoy mode: Revert; PSM FDR: 0.01; Protein FDR: 0.01; Min. peptide length for unspecific searches: 8; Max. peptide length for unspecific searches: 25; Peptides for quantification: Unique + razor; Use only unmodified peptides: True; Separate LFQ in parameter groups: false; Stabilize large LFQ ratios: True; Require MS/MS for LFQ comparisons: True; Missed cleavages: 2; Fixed modifications: nothing selected; Variable modifications: Oxidation (M) Carbamidomethyl (C) Deamination (NQ); Enzyme: Trypsin/P; Digestion mode: Semi-specific; Quantitation methods: LFQ; LFQ min. ratio count: 2; LFQ min. number of neighbours: 3; LFQ average number of neighbours: 6; Normalization type: Classic.

The Protein Groups file from the MaxQuant search was then input in LFQ Analyst (Dev.) (https://bioinformatics.erc.monash.edu/apps/LFQ-Analyst/, accessed 10 June 2023 [115]) for further analysis. LFQ analyst was set to 0.05 *p*-value cutoff, 1.5 log2 fold change cut off with Perseus-type imputation, no normalization and Benjamini–Hochberg FDR correction.

StringDB analysis was conducted using String V.11 using the following analysis parameters: Network type: Full string network; Meaning of network edges: evidence; Active interaction sources: Textmining, experiments, databases, co-expression, neighbourhood, gene fusion, co-occurrence; Minimum required interaction score: medium confidence (0.400); Max number of interactors to show: 1st shell—non/query proteins only. 2nd shell—none; Network display mode: interactive svg; and Network display options: disable 3D bubble design and disable structure previews inside network bubbles.

## Data Availability

The data presented in this study are available in Supplementary S1 and S2 and in supplementary material: String permalink with network functional analysis: https://version-11-5.string-db.org/cgi/network?networkId=bFY8QJcX4JxW accessed on 10 May 2023.

## Supplementary Materials

The following supporting information can be downloaded at: https://www.mdpi.com/article/10.3390/ijms241512139/s1.
