# Supplementary material for: Adipose-Derived Stem Cells Spontaneously Express Neural Markers When Grown in a PEG-Based 3D Matrix
Source: Int J Mol Sci. 2023 Jul 28;24(15):12139. doi: 10.3390/ijms241512139 (PMC10418654; doi:10.3390/ijms241512139)
Supplement: Supplementary file 1 [file ijms-24-12139-s001.zip › ijms-2512715-supplementary.pdf]

**Supplementary 1**

**Table S1** All proteins detected with significant change in abundance between treatments, including gene name, protein IDs, p-value and log fold change. Significant p-value < 0.05. The background colors refer to the change in abundance of each protein in the 3D culture as compared to the 2D culture. Increase in abundance was marked by blue, while decrease by red. The intensity of the color is proportional to the magnitude of each change (i.e. the darker the color the larger the fold change). Log2 scale was used.

| Gene Name | Protein IDs | 2D vs 3D Log2 fold change | 2D vs 3D p.value |
|---|---|---|---|
| CSRP1 | P21291 | 7.07 | 2.04E-10 |
| TIPRL | O75663 | 6.94 | 2.70E-08 |
| GLS.1 | O94925-3 | 5.5 | 1.01E-09 |
| SCAMP2 | O15127 | 5.15 | 3.40E-08 |
| ENPP1 | P22413 | 4.73 | 3.84E-05 |
| DSTN | P60981 | 4.72 | 0.000125 |
| CD63 | F8VNT9 | 4.7 | 1.23E-06 |
| NEK7 | Q8TDX7 | 4.52 | 8.38E-08 |
| PRNP | P04156 | 4.38 | 6.89E-06 |
| MCFD2 | Q8NI22 | 4.33 | 5.10E-06 |
| CTTN | Q14247 | 4.23 | 8.12E-07 |
| FAF2 | Q96CS3 | 4.17 | 0.000782 |
| PDAP1 | Q13442 | 4.13 | 1.09E-05 |
| LIMCH1 | Q9UPQ0 | 4.09 | 2.20E-07 |
| IAH1 | H7C5G1 | 3.84 | 1.47E-07 |
| DAB2 | P98082-3 | 3.79 | 7.33E-07 |
| AKR7A2 | O43488 | 3.77 | 9.47E-08 |
| MARCKS | P29966 | 3.74 | 8.22E-06 |
| AGFG1 | P52594-2 | 3.71 | 2.09E-08 |
| FAS | Q59FU8 | 3.69 | 7.85E-06 |
| HMGA1 | P17096 | 3.69 | 8.89E-08 |
| ERLIN2 | O94905 | 3.64 | 0.000133 |
| LMO7 | Q8WWI1-3 | 3.59 | 2.49E-07 |
| TAX1BP3 | O14907 | 3.57 | 1.94E-06 |
| SOD1 | P00441 | 3.56 | 0.000134 |
| HMOX2 | A0A087WT44 | 3.53 | 5.84E-06 |
| PHPT1 | Q9NRX4 | 3.53 | 5.60E-07 |
| BST1 | A6NC48 | 3.52 | 0.000386 |
| GRPEL1 | Q9HAV7 | 3.51 | 2.73E-06 |
| COPZ2 | Q9P299 | 3.48 | 4.13E-06 |
| ALYREF | E9PB61 | 3.47 | 0.00177 |
| CDH2 | C9J126 | 3.41 | 2.00E-05 |

| | | | |
|---|---|---|---|
| PIEZO1 | Q92508 | 3.41 | 1.30E-08 |
| NUTF2 | P61970 | 3.38 | 1.51E-05 |
| COL1A2 | P08123 | 3.32 | 7.96E-05 |
| EIF4H | A0A7I2V4E4 | 3.32 | 2.24E-05 |
| SORBS3 | O60504-2 | 3.3 | 5.08E-05 |
| CARHSP1 | Q9Y2V2 | 3.28 | 0.00373 |
| COL1A1 | P02452 | 3.28 | 5.09E-05 |
| LPXN | O60711 | 3.26 | 8.69E-06 |
| ABR | A0A0G2JQ41 | 3.23 | 8.23E-06 |
| KLC1 | G3V3H3 | 3.21 | 9.39E-05 |
| SH3GLB1 | A0A087WW40 | 3.2 | 6.71E-07 |
| DFFA | O00273 | 3.19 | 1.56E-06 |
| PSMD4 | Q5VWC4 | 3.18 | 2.76E-05 |
| CAP2 | P40123 | 3.16 | 1.77E-06 |
| EIF3E | P60228 | 3.15 | 0.00143 |
| OSTF1 | Q92882 | 3.14 | 1.68E-05 |
| GDI1 | P31150 | 3.13 | 1.85E-06 |
| UFC1 | Q9Y3C8 | 3.13 | 0.000964 |
| KHSRP | M0R0C6 | 3.09 | 2.25E-06 |
| TMEM119 | Q4V9L6 | 3.09 | 3.64E-07 |
| ANXA6 | E5RK69 | 3.08 | 2.27E-06 |
| MAP4K4 | E7ENQ1 | 3.08 | 1.22E-06 |
| RAB1A | P62820 | 3.08 | 6.81E-06 |
| TIMM9 | Q9Y5J7 | 3.08 | 1.53E-05 |
| RAPH1 | C9J164 | 3.05 | 2.85E-06 |
| PDHB | P11177-2 | 3.04 | 0.00117 |
| ACP2 | E9PQY3 | 3.03 | 0.000178 |
| PTK7 | Q13308-4 | 3.03 | 3.47E-07 |
| CALM2 | P0DP25 | 3.01 | 3.70E-06 |
| DDX6 | P26196 | 2.99 | 0.000504 |
| GRAMD3 | Q96HH9-4 | 2.98 | 2.03E-05 |
| ATP2B1 | P20020-1 | 2.96 | 4.13E-05 |
| TAGLN | Q01995 | 2.96 | 6.39E-07 |
| UBQLN2 | Q9UHD9 | 2.93 | 4.31E-05 |
| PDLIM2 | B3KPU0 | 2.91 | 0.00217 |
| CERCAM | Q5T4B2 | 2.89 | 0.000336 |
| AKAP12 | Q02952-3 | 2.88 | 4.90E-05 |
| RAI14 | Q9P0K7-4 | 2.87 | 0.000177 |
| TRIP6 | Q15654 | 2.85 | 2.86E-05 |
| TSNAX | Q99598 | 2.85 | 2.89E-05 |

| | | | |
|---|---|---|---|
| PRKRA | A0A7I2YQ87 | 2.81 | 0.00354 |
| NNMT | P40261 | 2.8 | 0.000381 |
| BAX | Q07812-5 | 2.79 | 0.00221 |
| ENG | P17813 | 2.79 | 1.45E-06 |
| SQSTM1 | Q13501 | 2.79 | 0.00264 |
| LOX | P28300 | 2.78 | 9.00E-04 |
| VAMP3 | Q15836 | 2.78 | 0.00274 |
| AHNAK2 | Q8IVF2 | 2.77 | 1.82E-05 |
| SERPINB1 | P30740 | 2.74 | 0.000122 |
| IFITM3 | E9PS44 | 2.72 | 2.02E-05 |
| DYNLT1 | P63172 | 2.71 | 0.000569 |
| PAFAH1B2 | P68402 | 2.71 | 3.57E-05 |
| LIMA1 | Q9UHB6 | 2.7 | 1.65E-05 |
| HN1 | J3KT51 | 2.69 | 0.000494 |
| SCUBE3 | Q8IX30 | 2.68 | 0.00155 |
| ATP5H | O75947 | 2.67 | 0.000542 |
| C21orf33 | P0DPI2 | 2.67 | 0.000689 |
| ADD1 | E7EV99 | 2.65 | 2.70E-05 |
| ZMPSTE24 | O75844 | 2.65 | 0.0116 |
| CLTA | P09496-2 | 2.64 | 0.000208 |
| PCBP1 | Q15365 | 2.64 | 0.000337 |
| PICALM | Q13492 | 2.64 | 0.000993 |
| CAPNS1 | A0A0C4DGQ5 | 2.61 | 0.000467 |
| CACYBP | Q9HB71 | 2.6 | 0.0012 |
| SLC30A1 | Q9Y6M5 | 2.6 | 0.000293 |
| TPD52L2 | O43399 | 2.6 | 0.00142 |
| BAG2 | O95816 | 2.58 | 7.84E-05 |
| RPL24 | C9JXB8 | 2.58 | 0.00102 |
| DLG1 | Q12959-5 | 2.56 | 4.64E-05 |
| GCC1 | Q96CN9 | 2.56 | 0.000677 |
| ITGA2 | P17301 | 2.55 | 0.0034 |
| FUBP3 | Q96I24 | 2.53 | 0.00157 |
| HERC4 | Q5GLZ8-3 | 2.53 | 0.000138 |
| C1orf198 | Q9H425-3 | 2.51 | 9.23E-06 |
| CTBP1 | Q13363-2 | 2.5 | 6.96E-05 |
| HSPB1 | P04792 | 2.5 | 2.72E-05 |
| ETFA | P13804 | 2.47 | 0.00338 |
| STAT3 | A0A7I2V4R2 | 2.44 | 8.69E-05 |
| TOLLIP | Q9H0E2 | 2.44 | 0.00157 |
| SLK | Q9H2G2 | 2.43 | 0.00527 |

| | | | |
|---|---|---|---|
| HSPB6 | O14558 | 2.42 | 0.000245 |
| PPP1R14B | Q96C90 | 2.42 | 0.000211 |
| PSMD5 | Q16401-2 | 2.42 | 0.000855 |
| SCYL1 | E9PS17 | 2.42 | 0.000139 |
| SORBS2 | O94875-11 | 2.42 | 0.000366 |
| SRP72 | O76094 | 2.42 | 0.00289 |
| BOLA2 | A0A499FJE1 | 2.4 | 0.00231 |
| HADHA | H0YFD6 | 2.4 | 2.77E-05 |
| PTRHD1 | Q6GMV3 | 2.4 | 0.00957 |
| STX4 | Q12846 | 2.4 | 9.44E-05 |
| AIMP2 | Q13155 | 2.39 | 0.00208 |
| PRRC1 | Q96M27 | 2.39 | 0.000461 |
| ZNF207 | J3QRS9 | 2.37 | 0.00142 |
| FKBP1A | P62942 | 2.35 | 7.70E-05 |
| LNP | Q9C0E8-2 | 2.34 | 0.00744 |
| CTNND1 | O60716 | 2.33 | 0.000223 |
| DCTD | P32321 | 2.33 | 0.000254 |
| HSPE1 | P61604 | 2.33 | 0.000215 |
| SPAG9 | O60271-4 | 2.33 | 0.00078 |
| ACAA1 | P09110 | 2.32 | 0.000599 |
| CMPK1 | P30085 | 2.32 | 5.52E-05 |
| IGBP1 | P78318 | 2.32 | 0.000239 |
| AP2A1 | O95782-2 | 2.31 | 0.00024 |
| FTL | P02792 | 2.31 | 7.59E-05 |
| PDLIM7 | Q9NR12 | 2.31 | 6.25E-05 |
| TBC1D2 | Q9BYX2-3 | 2.31 | 1.98E-05 |
| BANF1 | O75531 | 2.3 | 0.000285 |
| CTPS1 | A0A3B3IRI2 | 2.3 | 0.00158 |
| HIBADH | P31937 | 2.3 | 0.00562 |
| STK38L | Q9Y2H1 | 2.29 | 0.000483 |
| DDX42 | Q86XP3 | 2.28 | 9.12E-06 |
| SLC12A4 | Q9UP95 | 2.28 | 0.00167 |
| RAB5C | P51148 | 2.27 | 6.60E-05 |
| SYNPO2 | H0Y9Y3 | 2.27 | 0.00291 |
| SSSCA1 | G3V1B8 | 2.26 | 0.00407 |
| C1orf123 | Q9NWV4 | 2.25 | 0.00355 |
| TBCD | A0A804HLF8 | 2.24 | 0.000931 |
| AP2M1 | A0A8I5KWD3 | 2.23 | 0.00072 |
| GSS | A0A2R8Y430 | 2.23 | 0.0034 |
| CAPZA2 | P47755 | 2.21 | 1.83E-06 |

| | | | |
|---|---|---|---|
| COPS4 | Q9BT78 | 2.21 | 0.000208 |
| PPP1R9B | Q96SB3 | 2.21 | 0.000252 |
| RIC8A | Q9NPQ8-4 | 2.21 | 0.000118 |
| UBAP2L | Q14157-1 | 2.21 | 7.86E-05 |
| EMC8 | M0R1B0 | 2.2 | 0.00126 |
| PDXDC1 | H3BND4 | 2.2 | 1.10E-05 |
| PHLDB1 | Q86UU1-3 | 2.2 | 0.000192 |
| UBE2Z | Q9H832 | 2.2 | 0.00103 |
| NUDC | Q9Y266 | 2.19 | 0.0154 |
| MYL9 | P24844 | 2.18 | 9.36E-06 |
| RPS10 | P46783 | 2.18 | 0.00175 |
| TGM2 | P21980 | 2.18 | 3.36E-05 |
| API5 | Q9BZZ5-2 | 2.17 | 0.0125 |
| YKT6 | A0A7I2V4L6 | 2.16 | 0.000595 |
| CAV1 | Q03135 | 2.15 | 0.00094 |
| SNX1 | Q13596-2 | 2.15 | 0.00243 |
| CIRBP | Q14011 | 2.14 | 0.00104 |
| COMMD4 | A0A0B4J287 | 2.14 | 0.00628 |
| HSD17B10 | Q99714 | 2.14 | 0.00243 |
| RBM3 | P98179 | 2.14 | 0.0083 |
| TNS1 | A0A804HI61 | 2.13 | 0.000109 |
| TTLL12 | Q14166 | 2.12 | 0.00127 |
| AK4 | P27144 | 2.11 | 0.000339 |
| APPL2 | Q8NEU8 | 2.11 | 0.000262 |
| CCDC22 | O60826 | 2.11 | 3.89E-05 |
| CUL4B | A0A7P0T954 | 2.1 | 5.03E-05 |
| ARFGAP2 | Q8N6H7 | 2.09 | 0.00218 |
| EEF1B2 | P24534 | 2.09 | 2.67E-05 |
| ZYX | Q15942 | 2.09 | 0.000193 |
| APOOL | Q6UXV4 | 2.08 | 0.000161 |
| COPS3 | Q9UNS2-2 | 2.08 | 0.00041 |
| MAVS | Q7Z434 | 2.08 | 0.00287 |
| SEC24A | O95486 | 2.08 | 0.00284 |
| GLS | O94925 | 2.07 | 6.80E-06 |
| PPM1F | P49593-2 | 2.07 | 0.000203 |
| TBL2 | E9PF19 | 2.07 | 0.00245 |
| ECH1 | Q13011 | 2.06 | 0.0041 |
| DCTN2 | Q13561 | 2.04 | 0.0105 |
| IPO5 | O00410 | 2.04 | 2.42E-05 |
| RAB31 | Q13636 | 2.04 | 0.00134 |

| | | | |
|---|---|---|---|
| CAPZA1 | P52907 | 2.03 | 2.33E-05 |
| PURA | Q00577 | 2.03 | 0.0132 |
| CFL1 | E9PK25 | 2.02 | 0.00278 |
| IMMT.1 | Q16891-4 | 2.01 | 0.0111 |
| YBX3.1 | P16989-2 | 2.01 | 0.00642 |
| EHD1 | A0A024R571 | 2 | 4.49E-05 |
| EXOC2 | Q96KP1 | 2 | 0.000534 |
| B3GALTL | Q6Y288 | 1.99 | 0.000237 |
| YAP1 | P46937-5 | 1.99 | 0.00271 |
| MXRA7 | P84157-2 | 1.98 | 0.0046 |
| TMPO | P42167 | 1.98 | 9.51E-05 |
| EXOC1 | Q9NV70-2 | 1.95 | 0.000637 |
| SH3BGRL3 | Q9H299 | 1.95 | 9.83E-05 |
| SNAP23 | O00161 | 1.95 | 0.00287 |
| CYBRD1 | Q53TN4-3 | 1.94 | 0.00369 |
| SCAMP1 | A0A087WXB0 | 1.94 | 0.00277 |
| SMS | P52788 | 1.94 | 0.0114 |
| C14orf166 | Q9Y224 | 1.93 | 7.27E-05 |
| CACNA2D1 | P54289-4 | 1.93 | 0.00105 |
| MRPL50 | Q8N5N7 | 1.93 | 0.00123 |
| TMEM214 | Q6NUQ4 | 1.93 | 0.00558 |
| UBXN6 | Q9BZV1-2 | 1.92 | 0.00628 |
| PAWR | Q96IZ0 | 1.91 | 0.000362 |
| C12orf75 | F8VQD4 | 1.9 | 0.000997 |
| EIF3F | H0YDT6 | 1.9 | 0.00022 |
| PPME1 | Q9Y570 | 1.89 | 0.00147 |
| CD81 | E9PJK1 | 1.88 | 8.02E-05 |
| CUL4A | Q13619 | 1.88 | 0.00215 |
| FXR2 | P51116 | 1.88 | 3.08E-05 |
| ALCAM | Q13740 | 1.87 | 0.000546 |
| PDCD5 | O14737 | 1.87 | 0.00585 |
| PFDN2 | Q9UHV9 | 1.87 | 0.00142 |
| LAMTOR3 | Q9UHA4-2 | 1.86 | 0.00626 |
| ZC3HAV1 | Q7Z2W4 | 1.86 | 0.00783 |
| CARS | P49589 | 1.85 | 0.000213 |
| PRPSAP1 | Q14558 | 1.85 | 0.00261 |
| STRN | O43815-2 | 1.84 | 0.000332 |
| IMMT | Q16891-2 | 1.83 | 7.60E-05 |
| M6PR | H0YGT2 | 1.83 | 0.00831 |
| PRKCDBP | Q969G5 | 1.83 | 0.000896 |

| | | | |
|---|---|---|---|
| GSK3B | P49841 | 1.82 | 0.000612 |
| UNC45A | A0A1W2PNX8 | 1.82 | 0.0039 |
| LAMTOR1 | Q6IAA8 | 1.8 | 0.00102 |
| WDR44 | Q5JSH3-4 | 1.78 | 0.00393 |
| DBNL | Q9UJU6 | 1.77 | 0.000856 |
| GALK1 | P51570 | 1.77 | 0.00291 |
| SFXN1 | Q9H9B4 | 1.77 | 0.000734 |
| ATP6V1C1 | P21283 | 1.76 | 0.000381 |
| MT-CO2 | P00403 | 1.76 | 9.58E-05 |
| EEF1D | P29692-2 | 1.75 | 0.000316 |
| ERGIC1 | Q969X5-2 | 1.75 | 0.000905 |
| CCDC80 | Q76M96-2 | 1.74 | 0.000206 |
| EIF3M | Q7L2H7 | 1.73 | 0.00746 |
| PAICS | P22234 | 1.73 | 0.000102 |
| PEA15 | Q15121 | 1.73 | 0.000334 |
| AAK1 | A0A096LP25 | 1.72 | 0.000274 |
| ARSB | P15848 | 1.72 | 0.00134 |
| SPATS2L | B8ZZZ7 | 1.72 | 3.33E-05 |
| PDIA6 | Q15084-3 | 1.71 | 0.000393 |
| ASAP2 | O43150 | 1.7 | 0.00287 |
| PNN | Q9H307 | 1.7 | 0.00329 |
| SEC16A | A0A8I5KPG1 | 1.7 | 0.00186 |
| GYG1 | P46976-2 | 1.69 | 6.50E-05 |
| HSPB2 | Q16082 | 1.69 | 0.00704 |
| TAGLN2 | P37802 | 1.69 | 0.00025 |
| UQCRC1 | P31930 | 1.69 | 0.00166 |
| BAG3 | O95817 | 1.68 | 0.00106 |
| TUBB4A | P04350 | 1.68 | 0.0106 |
| CORO1C | Q9ULV4-3 | 1.67 | 0.00221 |
| NPM1 | P06748 | 1.67 | 0.00415 |
| UAP1 | Q16222-3 | 1.67 | 0.000117 |
| NEU1 | Q99519 | 1.66 | 0.00266 |
| PLIN3 | O60664 | 1.66 | 0.00336 |
| CKAP5 | Q14008-2 | 1.65 | 0.00796 |
| STXBP1 | A0A1B0GWF2 | 1.63 | 0.00113 |
| BSG | P35613-2 | 1.62 | 0.000908 |
| CLTB | P09497-2 | 1.62 | 0.000672 |
| CNN2 | B4DDF4 | 1.62 | 0.000675 |
| PGM1 | A0A3B3ITK7 | 1.62 | 0.00317 |
| ARHGEF10 | H0YAN8 | 1.61 | 0.00596 |

| | | | |
|---|---|---|---|
| IFI16 | Q16666-3 | 1.61 | 0.00684 |
| PPM1G | O15355 | 1.61 | 0.0136 |
| STAT1 | P42224 | 1.61 | 0.000129 |
| UBA1 | P22314-2 | 1.61 | 0.000278 |
| THY1 | E9PIM6 | 1.6 | 0.00011 |
| TWF2 | Q6IBS0 | 1.6 | 0.000602 |
| CCT2 | P78371 | 1.59 | 0.00029 |
| DBN1 | Q16643 | 1.59 | 0.00025 |
| EPB41L2 | O43491 | 1.59 | 0.00107 |
| HNRNPM | A0A087X0X3 | 1.59 | 0.000119 |
| LAMP2 | P13473-2 | 1.59 | 0.00949 |
| CSRP2 | Q16527 | 1.58 | 0.000413 |
| HDGFRP2 | Q7Z4V5-2 | 1.58 | 0.00308 |
| DNAJB1 | M0QXK0 | 1.57 | 0.000299 |
| ENDOD1 | O94919 | 1.57 | 0.00077 |
| S100A10 | P60903 | 1.57 | 0.000783 |
| CCT6A | P40227 | 1.56 | 0.00286 |
| DDX19A | I3L0H8 | 1.56 | 0.0013 |
| NDRG1 | Q92597 | 1.56 | 0.00143 |
| PPP3CA | Q08209-3 | 1.56 | 0.015 |
| SH3KBP1 | Q5JPT2 | 1.56 | 0.013 |
| COL5A2 | P05997 | 1.54 | 0.00646 |
| ARL6IP5 | O75915 | 1.53 | 0.00165 |
| PPP1R18 | A0A0G2JHC2 | 1.53 | 0.00208 |
| CMAS | Q8NFW8 | 1.52 | 0.0118 |
| MYL12B | O14950 | 1.52 | 0.000426 |
| C9orf89 | Q96LW7-2 | 1.51 | 0.00294 |
| SEMA7A | O75326 | 1.51 | 0.00074 |
| TUBB3 | Q13509 | 1.51 | 0.000672 |
| HIST1H3A | P68431 | -1.55 | 0.0144 |
| PSPH | C9JBI3 | -1.55 | 0.00195 |
| ASMTL | O95671-3 | -1.57 | 0.00237 |
| CCAR1 | Q8IX12-2 | -1.57 | 0.00879 |
| CRLF1 | O75462 | -1.57 | 0.00251 |
| HSD17B12 | Q53GQ0 | -1.59 | 0.000201 |
| SAR1A | Q9NR31 | -1.59 | 0.000988 |
| PSMB5 | P28074 | -1.63 | 0.00166 |
| UBTF | E9PKP7 | -1.64 | 0.00808 |
| NAA25 | Q14CX7-2 | -1.66 | 0.011 |
| SYNE3 | Q6ZMZ3-2 | -1.66 | 0.00151 |

| | | | |
|---|---|---|---|
| NDUFA9 | Q16795 | -1.67 | 0.00323 |
| RRAGD | Q9NQL2-2 | -1.69 | 0.00311 |
| JUP | P14923 | -1.71 | 0.00756 |
| SUN2 | B0QY64 | -1.71 | 0.000302 |
| FXR1 | E7EU85 | -1.72 | 0.000139 |
| TUBB8 | Q3ZCM7 | -1.72 | 0.00576 |
| TPM4 | P67936 | -1.73 | 0.000104 |
| ARPC5L | Q9BPX5 | -1.76 | 0.0146 |
| PRKG1 | Q13976 | -1.76 | 0.00216 |
| RPS4X | P62701 | -1.76 | 0.0123 |
| SERBP1 | Q8NC51 | -1.77 | 0.009 |
| COG2 | B7Z2Y2 | -1.79 | 0.00576 |
| UQCRFS1 | P47985 | -1.79 | 0.00218 |
| PSAT1 | Q9Y617 | -1.8 | 0.000223 |
| RPL23A | P62750 | -1.8 | 0.00177 |
| TMED10 | P49755 | -1.8 | 0.000361 |
| HIST1H4A | P62805 | -1.81 | 0.000599 |
| PRMT5 | O14744-2 | -1.81 | 0.00406 |
| EIF4B | E7EX17 | -1.84 | 0.000388 |
| NDUFC2 | O95298-2 | -1.85 | 0.00288 |
| BLMH | Q13867 | -1.86 | 0.000498 |
| COL6A3 | P12111-2 | -1.86 | 0.000102 |
| BASP1 | P80723 | -1.87 | 0.00297 |
| FKBP9 | O95302 | -1.87 | 0.00153 |
| NQO1 | P15559-2 | -1.89 | 0.000525 |
| RPS5 | M0R0F0 | -1.89 | 0.00413 |
| SRSF3 | P84103 | -1.89 | 0.00113 |
| PTGES3L-AARSD1 | C9J5N1 | -1.92 | 4.92E-05 |
| PWP1 | Q13610 | -1.92 | 0.0033 |
| RECQL | P46063 | -1.92 | 0.00262 |
| TMED4 | Q7Z7H5-3 | -1.93 | 0.000142 |
| DCPS | Q96C86 | -1.94 | 0.00128 |
| FKBP7 | Q9Y680-3 | -1.94 | 0.00123 |
| LMNB1 | P20700 | -1.94 | 9.98E-05 |
| TJP1 | A0A0G2JNH2 | -1.96 | 0.00135 |
| CDH13 | P55290 | -1.99 | 0.000522 |
| CCDC47 | Q96A33 | -2.02 | 0.00808 |
| SNX18 | Q96RF0-2 | -2.02 | 0.000751 |
| SCPEP1 | Q9HB40 | -2.03 | 6.86E-05 |
| YWHAE | P62258 | -2.03 | 0.00024 |

| | | | |
|---|---|---|---|
| SRRT | Q9BXP5-5 | -2.07 | 0.0051 |
| SF1 | A0A7P0T9U7 | -2.09 | 0.00103 |
| DHX29 | Q7Z478 | -2.1 | 0.000199 |
| RASA4B | F8W6L0 | -2.11 | 0.00161 |
| RSL1D1 | J3QSV6 | -2.12 | 0.000383 |
| BZW1 | C9IZ80 | -2.15 | 0.00918 |
| CTSL | P07711-3 | -2.16 | 8.41E-05 |
| PSMA7 | O14818 | -2.16 | 0.000164 |
| RPL15 | A0A2R8YEM3 | -2.18 | 0.00263 |
| UBLCP1 | Q8WVY7 | -2.2 | 0.000385 |
| EIF1AX | P47813 | -2.21 | 5.91E-06 |
| SLC16A3 | J3QQV2 | -2.22 | 5.39E-05 |
| LRRFIP2 | Q9Y608-4 | -2.24 | 0.00242 |
| SZRD1 | Q7Z422-2 | -2.24 | 5.74E-05 |
| ACSL3 | A0A7P0TA76 | -2.27 | 0.00529 |
| ENAH | Q8N8S7-3 | -2.27 | 0.00426 |
| SNRPD1 | J3QLI9 | -2.28 | 0.00488 |
| UBE2K | P61086 | -2.28 | 0.00305 |
| CRYZ | Q08257 | -2.29 | 0.00464 |
| RANBP1 | P43487-2 | -2.3 | 0.000726 |
| IDE | A0A7I2V3E3 | -2.31 | 6.96E-05 |
| MAGOHB | F5H6N1 | -2.31 | 0.0104 |
| MAN2A1 | Q16706 | -2.33 | 0.000289 |
| PRKAG1 | P54619-3 | -2.35 | 0.00763 |
| HIST1H1E | P10412 | -2.39 | 2.28E-05 |
| ILVBL | A1L0T0 | -2.4 | 0.00474 |
| RPS11 | P62280 | -2.41 | 0.00247 |
| FAU | E9PR30 | -2.43 | 0.000207 |
| COL15A1 | A0A087X0K0 | -2.45 | 0.00404 |
| HTRA1 | Q92743 | -2.49 | 0.0109 |
| MTX1 | Q13505-3 | -2.49 | 6.25E-06 |
| PSMB6 | P28072 | -2.49 | 2.09E-05 |
| HSDL2 | Q6YN16-2 | -2.54 | 6.62E-05 |
| MGST3 | O14880 | -2.54 | 0.0146 |
| NQO2 | Q5TD07 | -2.54 | 0.00577 |
| PPT1 | A0A2C9F2P4 | -2.59 | 0.000345 |
| RDX | A0A2R8Y5S7 | -2.59 | 7.07E-05 |
| RPS6 | P62753 | -2.63 | 2.51E-05 |
| SSR2 | P43308 | -2.65 | 0.00891 |
| WDR61 | H0YN81 | -2.7 | 6.06E-05 |

| | | | |
|---|---|---|---|
| RPA1 | P27694 | -2.71 | 7.03E-05 |
| UBE2I | H3BQQ9 | -2.71 | 0.0115 |
| AKR1C2 | P52895 | -2.72 | 3.39E-05 |
| RPL35 | P42766 | -2.79 | 6.63E-05 |
| NIPSNAP3A | Q9UFN0 | -2.82 | 1.20E-05 |
| LGMN | Q99538-3 | -2.83 | 0.000155 |
| NACA | F8W0W4 | -2.83 | 0.000127 |
| MTHFD2 | A0A7I2V2U6 | -2.87 | 0.00146 |
| PSMA2 | A0A024RA52 | -2.87 | 0.0101 |
| LUM | P51884 | -2.97 | 0.00128 |
| EWSR1 | B0QYK0 | -2.98 | 0.000228 |
| NOP56 | O00567 | -2.99 | 7.79E-06 |
| PEX11B | O96011 | -2.99 | 2.05E-06 |
| CD151 | K4DIA7 | -3.01 | 0.000345 |
| SEC61A1 | B4DR61 | -3.03 | 9.06E-07 |
| GAP43 | P17677 | -3.08 | 6.30E-06 |
| SRP68 | Q9UHB9-4 | -3.09 | 8.62E-05 |
| PSMA3 | P25788-2 | -3.1 | 3.11E-05 |
| GSTM3 | P21266 | -3.12 | 0.00448 |
| DDX21 | A0A8I5KNP3 | -3.14 | 6.13E-06 |
| SELM | Q8WWX9 | -3.16 | 0.000812 |
| RPL13a | Q8J015 | -3.24 | 5.71E-05 |
| H2AFV | Q71UI9 | -3.26 | 0.00389 |
| ALDH3A2 | P51648-2 | -3.29 | 1.02E-05 |
| RBBP7 | E9PC52 | -3.3 | 1.11E-05 |
| ACAT2 | Q9BWD1 | -3.52 | 2.43E-05 |
| COMP | G3XAP6 | -3.59 | 0.00399 |
| TMSB10 | P63313 | -3.59 | 0.000222 |
| SDCBP | O00560-3 | -3.61 | 0.000492 |
| RPL26 | P61254 | -3.64 | 1.58E-06 |
| RPS15 | K7ELC2 | -3.65 | 2.00E-05 |
| XXYLT1 | A0A140T9D0 | -3.71 | 0.00101 |
| B2M | P61769 | -3.87 | 4.93E-06 |
| ATP5D | P30049 | -3.92 | 2.16E-06 |
| PPIC | P45877 | -3.94 | 1.54E-05 |
| CD276 | A0A0C4DGH0 | -3.95 | 1.31E-06 |
| RPLP1 | P05386 | -4.02 | 0.000912 |
| ARF5 | P84085 | -4.04 | 4.50E-07 |
| MAP1LC3B | H3BTL1 | -4.29 | 7.26E-08 |
| HIST2H3PS2 | Q5TEC6 | -4.33 | 0.00262 |

| | | | |
|---|---|---|---|
| RBBP4 | Q09028-4 | -4.56 | 2.51E-07 |
| EIF6 | P56537 | -4.58 | 0.000345 |
| KRT6B | CON_P04259 | -4.7 | 0.00328 |
| RSU1 | Q15404 | -5.15 | 5.52E-08 |
| UBE2D3 | P61077-2 | -5.8 | 3.52E-07 |
| TMSB4X | P62328 | -6.28 | 2.63E-08 |
| BTF3 | P20290-2 | -6.61 | 6.64E-08 |

## Supplementary S2

**Table S2.** All proteins involved in processes and functions of actin, ribosomal and neural. including fold change. With blue representing an increase in abundance in 3D compared to 2D and red representing a decrease in abundance in 3D compared to 2D.

| Protein | Log2 Fold change | Functions | Involvement |
|---|---|---|---|
| DAB2 | 3.79 | Actin cytoskeleton | actin |
| TAX1BP3 | 3.57 | Actin cytoskeleton, Actin cytoskeleton | actin |
| BST1 | 3.52 | Regulation of actin filament-based process, Regulation of actin cytoskeleton organization | actin |
| LPXN | 3.26 | Actin cytoskeleton, Actin cytoskeleton | actin |
| TAGLN | 2.96 | Actin binding, Actin filament binding, Actin-binding | actin |
| PDLIM2 | 2.91 | Actin cytoskeleton organization, Actin filament-based process, Actin binding, Actin cytoskeleton, Actin filament bundle, Mixed, incl. actin filament organization, and filamentous actin | actin |
| TRIP6 | 2.85 | Actin cytoskeleton, Actin filament bundle, Actin cytoskeleton, Actin filament bundle | actin |
| STK38L | 2.29 | Actin binding, Actin cytoskeleton, Actin cytoskeleton, Actin-binding | actin |
| SYNPO2 | 2.27 | Regulation of actin filament-based process, Regulation of actin filament organization, Regulation of actin cytoskeleton organization, Actin binding, Actin cytoskeleton, Actin filament bundle, Actin cytoskeleton, Actin filament bundle, Actin-binding | actin |
| TNS1 | 2.13 | Actin binding, Mixed, incl. actin filament organization, and filamentous actin, Mixed, incl. regulation of actin polymerization or depolymerization, and Calponin homology (CH) domain, RHO GTPases Activate WASPs and WAVEs, and actin filament organization, Actin-binding | actin |
| ZYX | 2.09 | Actin cytoskeleton organization, Actin filament-based process, Actin filament organization, Actin cytoskeleton, Actin filament bundle, Mixed, incl. actin filament organization, and filamentous actin, Mixed, incl. regulation of actin polymerization or depolymerization, and Calponin homology (CH) domain, RHO GTPases Activate WASPs and WAVEs, and actin filament organization, Actin cytoskeleton, Actin filament bundle | actin |
| CAPZA1 | 2.03 | Actin cytoskeleton organization, Actin filament-based process, Regulation of actin filament-based process, Regulation of actin filament organization, Regulation of actin cytoskeleton organization, Regulation of actin filament depolymerization, Regulation of actin polymerization or depolymerization, Barbed-end actin filament capping, Negative regulation of actin filament depolymerization, Negative regulation of actin filament polymerization, Regulation of actin filament polymerization, Actin binding, Actin filament binding, Actin cytoskeleton, F-actin capping protein complex, Actin cytoskeleton, F-actin capping protein complex, Actin-binding | actin |

| | | | |
|---|---|---|---|
| PAWR | 1.91 | Actin cytoskeleton organization, Actin filament-based process, Actin filament organization, Actin binding, Actin cytoskeleton, Actin cytoskeleton | actin |
| PRKCDBP | 1.83 | Actin cytoskeleton organization, Actin filament-based process | actin |
| TAGLN2 | 1.69 | Mixed, incl. actin filament organization, and filamentous actin, Mixed, incl. regulation of actin polymerization or depolymerization, and Calponin homology (CH) domain, RHO GTPases Activate WASPs and WAVEs, and actin filament organization | actin |
| CNN2 | 1.62 | Actin cytoskeleton organization, Actin filament-based process, Regulation of actin filament-based process, Actin binding, Actin cytoskeleton, Actin filament bundle, Actin cytoskeleton, Actin filament bundle, Actin-binding | actin |
| CSRP2 | 1.58 | Actin cytoskeleton organization, Actin filament-based process | actin |
| PPP1R18 | 1.53 | Actin binding, Actin-binding | actin |
| SYNE3 | -1.66 | Actin binding, Actin filament binding | actin |
| JUP | -1.71 | Regulation of actin filament-based process, Actin cytoskeleton | actin |
| PRKG1 | -1.76 | Actin cytoskeleton organization, Actin filament-based process | actin |
| CSRP1 | 7.07 | Actin cytoskeleton organization, Actin filament-based process, Nervous system, Central nervous system, Brain, Forebrain | actin, neural |
| DSTN | 4.72 | Actin cytoskeleton organization, Actin filament-based process, Actin filament organization, Regulation of actin filament-based process, Regulation of actin filament organization, Regulation of actin cytoskeleton organization, Regulation of actin filament depolymerization, Regulation of actin polymerization or depolymerization, Actin filament depolymerization, Actin polymerization or depolymerization, Actin binding, Actin filament binding, Actin cytoskeleton, Cortical actin cytoskeleton, Mixed, incl. actin filament organization, and filamentous actin, Mixed, incl. regulation of actin polymerization or depolymerization, and Calponin homology (CH) domain, RHO GTPases Activate WASPs and WAVEs, and actin filament organization, Actin-binding, Nervous system, Central nervous system, Brain | actin, neural |
| CTTN | 4.23 | Actin cytoskeleton organization, Actin filament-based process, Actin filament organization, Regulation of actin filament-based process, Regulation of actin filament organization, Regulation of actin cytoskeleton organization, Regulation of actin polymerization or depolymerization, Actin polymerization or depolymerization, Regulation of actin filament polymerization, Actin binding, Actin filament binding, Actin cytoskeleton, Cortical actin cytoskeleton, Actin cytoskeleton, Positive regulation of axon extension, Neuron projection, Somatodendritic compartment, Dendrite, Postsynapse organization, Synapse, Postsynaptic density, Postsynapse | actin, neural |

| | | | |
|---|---|---|---|
| LIMCH1 | 4.09 | Actin cytoskeleton organization, Actin filament-based process, Regulation of actin filament-based process, Regulation of actin filament organization, Regulation of actin cytoskeleton organization, Actin binding, Actin cytoskeleton, Actin filament bundle, Actin cytoskeleton, Actin filament bundle, Nervous system, Central nervous system, Brain | actin, neural |
| MARCKS | 3.74 | Actin cytoskeleton organization, Actin filament-based process, Actin filament organization, Actin binding, Actin filament binding, Actin cytoskeleton, Actin filament bundle, Actin cytoskeleton, Actin-binding, Nervous system, Central nervous system, Brain | actin, neural |
| PHPT1 | 3.53 | Regulation of actin filament-based process, Regulation of actin cytoskeleton organization, Nervous system, Central nervous system, Brain | actin, neural |
| SORBS3 | 3.3 | Actin cytoskeleton organization, Actin filament-based process, Actin filament organization, Regulation of actin filament-based process, Regulation of actin filament organization, Regulation of actin cytoskeleton organization, Nervous system | actin, neural |
| ABR | 3.23 | Actin cytoskeleton organization, Actin filament-based process, Axon, Neuron projection, Somatodendritic compartment, Dendrite, Synapse, Postsynaptic density, Postsynapse, Glutamatergic synapse, Nervous system, Central nervous system, Brain, Forebrain, Cerebral cortex | actin, neural |
| CAP2 | 3.16 | Actin cytoskeleton organization, Actin filament-based process, Actin filament organization, Actin polymerization or depolymerization, Actin binding, Actin cytoskeleton, Cortical actin cytoskeleton, Mixed, incl. actin filament organization, and filamentous actin, Mixed, incl. regulation of actin polymerization or depolymerization, and Calponin homology (CH) domain, RHO GTPases Activate WASPs and WAVEs, and actin filament organization, Axon guidance, Synapse, Postsynaptic density, Postsynapse, Nervous system development, Nervous system, Central nervous system, Brain, Forebrain, Cerebral cortex, Amygdala, Basal ganglion, Ganglion | actin, neural |
| ANXA6 | 3.08 | Actin binding, Actin filament binding, Nervous system, Central nervous system, Brain, Forebrain, Cerebral cortex, Amygdala, Basal ganglion, Ganglion | actin, neural |
| PTK7 | 3.03 | Actin cytoskeleton organization, Actin filament-based process, Wnt signaling pathway, planar cell polarity pathway | actin, neural |
| RAI14 | 2.87 | Actin binding, Nervous system, Central nervous system, Brain | actin, neural |

| | | | |
|---|---|---|---|
| LIMA1 | 2.7 | Actin cytoskeleton organization, Actin filament-based process, Actin filament organization, Regulation of actin filament-based process, Regulation of actin filament organization, Regulation of actin cytoskeleton organization, Regulation of actin filament depolymerization, Regulation of actin polymerization or depolymerization, Negative regulation of actin filament depolymerization, Actin binding, Actin filament binding, Actin cytoskeleton, Actin filament bundle, Actin cytoskeleton, Actin filament bundle, Actin-binding, Nervous system, Central nervous system, Brain, Forebrain | actin, neural |
| ADD1 | 2.65 | Actin cytoskeleton organization, Actin filament-based process, Actin filament organization, Regulation of actin filament-based process, Regulation of actin filament organization, Regulation of actin cytoskeleton organization, Regulation of actin filament depolymerization, Regulation of actin polymerization or depolymerization, Barbed-end actin filament capping, Negative regulation of actin filament depolymerization, Negative regulation of actin filament polymerization, Regulation of actin filament polymerization, Actin binding, Actin filament binding, Actin cytoskeleton, F-actin capping protein complex, Actin cytoskeleton, F-actin capping protein complex, Actin-binding, Synapse, Postsynaptic density, Postsynapse, Nervous system, Central nervous system, Brain, Forebrain, Cerebral cortex | actin, neural |
| DLG1 | 2.56 | Actin cytoskeleton organization, Actin filament-based process, Actin filament organization, Regulation of actin filament-based process, Regulation of actin filament organization, Regulation of actin cytoskeleton organization, Regulation of actin polymerization or depolymerization, Regulation of actin filament polymerization, Axon, Axon guidance, Neuron projection, Postsynapse organization, Synapse, Postsynaptic density, Postsynapse, Glutamatergic synapse, Nervous system development, Nervous system, Central nervous system, Brain | actin, neural |
| SORBS2 | 2.42 | Actin cytoskeleton organization, Actin filament-based process, Actin filament organization, Actin cytoskeleton, Actin cytoskeleton, Nervous system, Central nervous system, Brain, Forebrain | actin, neural |
| PDLIM7 | 2.31 | Actin cytoskeleton organization, Actin filament-based process, Actin binding, Actin cytoskeleton, Actin filament bundle, Mixed, incl. actin filament organization, and filamentous actin, Actin cytoskeleton, Axon guidance, Nervous system development, Nervous system, Central nervous system, Brain | actin, neural |

| | | | |
|---|---|---|---|
| CAPZA2 | 2.21 | Actin cytoskeleton organization, Actin filament-based process, Regulation of actin filament-based process, Regulation of actin filament organization, Regulation of actin cytoskeleton organization, Regulation of actin filament depolymerization, Regulation of actin polymerization or depolymerization, Barbed-end actin filament capping, Negative regulation of actin filament depolymerization, Negative regulation of actin filament polymerization, Regulation of actin filament polymerization, Actin binding, Actin filament binding, Actin cytoskeleton, F-actin capping protein complex, Actin cytoskeleton, F-actin capping protein complex, Actin-binding, Nervous system, Central nervous system, Brain, Forebrain, Cerebral cortex, Amygdala, Basal ganglion, Ganglion | actin, neural |
| PPP1R9B | 2.21 | Actin cytoskeleton organization, Actin filament-based process, Actin filament organization, Actin filament depolymerization, Actin polymerization or depolymerization, Actin binding, Actin filament binding, Actin cytoskeleton, Cortical actin cytoskeleton, Actin-binding, Filopodium, Filopodium, Growth cone, Axon, Distal axon, Neuron projection, Neuronal cell body, Somatodendritic compartment, Dendritic spine neck, Dendrite, Synapse, Postsynaptic density, Postsynapse, Nervous system, Central nervous system, Brain, Forebrain, Cerebral cortex, Amygdala, Basal ganglion, Ganglion | actin, neural |
| MYL9 | 2.18 | Actin cytoskeleton, Actin filament bundle, Actin cytoskeleton, Axon guidance, Nervous system development | actin, neural |
| CAV1 | 2.15 | Regulation of actin filament-based process, Nervous system, Central nervous system, Brain | actin, neural |
| PPM1F | 2.07 | Regulation of actin filament-based process, Regulation of actin filament organization, Regulation of actin cytoskeleton organization, Nervous system, Central nervous system, Brain | actin, neural |
| DCTN2 | 2.04 | Actin cytoskeleton, Actin cytoskeleton, Growth cone, Axon, Distal axon, Neuron projection, Nervous system, Central nervous system, Brain | actin, neural |
| CFL1 | 2.02 | Actin cytoskeleton organization, Actin filament-based process, Actin filament organization, Regulation of actin filament-based process, Regulation of actin filament organization, Regulation of actin cytoskeleton organization, Regulation of actin filament depolymerization, Regulation of actin polymerization or depolymerization, Actin filament depolymerization, Actin polymerization or depolymerization, Actin binding, Actin filament binding, Actin cytoskeleton, Cortical actin cytoskeleton, Mixed, incl. actin filament organization, and filamentous actin, Mixed, incl. regulation of actin polymerization or depolymerization, and Calponin homology (CH) domain, Actin cytoskeleton, Actin filament bundle, Actin-binding, Axon guidance, Nervous system development, Nervous system, Central nervous system, Brain, Brain cell line | actin, neural |
| CACNA2D1 | 1.93 | Actin filament-based process, Nervous system, Central nervous system, Brain | actin, neural |

| | | | |
|---|---|---|---|
| DBNL | 1.77 | Actin cytoskeleton organization, Actin filament-based process, Regulation of actin filament-based process, Regulation of actin filament organization, Regulation of actin cytoskeleton organization, Regulation of actin polymerization or depolymerization, Regulation of actin filament polymerization, Actin binding, Actin filament binding, Actin cytoskeleton, Cortical actin cytoskeleton, Actin cytoskeleton, Actin-binding, Positive regulation of axon extension, Neuron projection, Neuronal cell body, Somatodendritic compartment, Dendrite, Postsynapse organization, Synapse, Postsynaptic density, Postsynapse, Nervous system, Central nervous system, Brain | actin, neural |
| BAG3 | 1.68 | Actin cytoskeleton, Actin filament bundle, Neuron projection, Nervous system, Central nervous system, Brain | actin, neural |
| CORO1C | 1.67 | Actin cytoskeleton organization, Actin filament-based process, Actin filament organization, Actin binding, Actin filament binding, Actin cytoskeleton, Mixed, incl. actin filament organization, and filamentous actin, Mixed, incl. regulation of actin polymerization or depolymerization, and Calponin homology (CH) domain, RHO GTPases Activate WASPs and WAVEs, and actin filament organization, Actin cytoskeleton, Actin-binding, Synapse, Nervous system, Central nervous system, Brain | actin, neural |
| ARHGEF10 | 1.61 | Actin cytoskeleton organization, Actin filament-based process, Regulation of actin filament-based process, Regulation of actin filament organization, Regulation of actin cytoskeleton organization, Nervous system, Central nervous system, Brain | actin, neural |
| TWF2 | 1.6 | Actin cytoskeleton organization, Actin filament-based process, Actin filament organization, Regulation of actin filament-based process, Regulation of actin filament organization, Regulation of actin cytoskeleton organization, Regulation of actin filament depolymerization, Regulation of actin polymerization or depolymerization, Barbed-end actin filament capping, Actin filament depolymerization, Negative regulation of actin filament depolymerization, Negative regulation of actin filament polymerization, Actin polymerization or depolymerization, Regulation of actin filament polymerization, Actin binding, Actin filament binding, Actin cytoskeleton, Mixed, incl. actin filament organization, and filamentous actin, Mixed, incl. regulation of actin polymerization or depolymerization, and Calponin homology (CH) domain, RHO GTPases Activate WASPs and WAVEs, and actin filament organization, Actin-binding, Filopodium, Growth cone, Positive regulation of axon extension, Axon, Distal axon, Neuron projection | actin, neural |

| | | | |
|---|---|---|---|
| DBN1 | 1.59 | Actin cytoskeleton organization, Actin filament-based process, Actin filament organization, Regulation of actin filament-based process, Regulation of actin filament organization, Regulation of actin cytoskeleton organization, Regulation of actin polymerization or depolymerization, Regulation of actin filament polymerization, Actin binding, Actin filament binding, Actin cytoskeleton, Cortical actin cytoskeleton, Mixed, incl. actin filament organization, and filamentous actin, Actin cytoskeleton, Actin-binding, Growth cone, Positive regulation of axon extension, Axon, Distal axon, Neuron projection, Somatodendritic compartment, Dendrite, Postsynapse organization, Synapse, Postsynaptic density, Postsynapse, Glutamatergic synapse, Nervous system, Central nervous system, Brain | actin, neural |
| EPB41L2 | 1.59 | Actin cytoskeleton organization, Actin filament-based process, Actin binding, Actin cytoskeleton, Cortical actin cytoskeleton, Actin cytoskeleton, Actin-binding, Nervous system, Central nervous system, Brain | actin, neural |
| ENDOD1 | 1.57 | Mixed, incl. actin filament organization, and filamentous actin, Mixed, incl. regulation of actin polymerization or depolymerization, and Calponin homology (CH) domain, RHO GTPases Activate WASPs and WAVEs, and actin filament organization, Nervous system, Central nervous system, Brain | actin, neural |
| S100A10 | 1.57 | Regulation of actin filament-based process, Regulation of actin filament organization, Regulation of actin cytoskeleton organization, Nervous system, Central nervous system, Brain | actin, neural |
| SH3KBP1 | 1.56 | Actin cytoskeleton organization, Actin filament-based process, Actin filament organization, Axon guidance, Neuron projection, Synapse, Nervous system development | actin, neural |
| MYL12B | 1.52 | Actin cytoskeleton, Actin filament bundle, Actin cytoskeleton, Axon guidance, Nervous system development, Nervous system, Central nervous system, Brain | actin, neural |
| SUN2 | -1.71 | Actin filament-based process, Nervous system, Central nervous system, Brain | actin, neural |
| TPM4 | -1.73 | Actin cytoskeleton organization, Actin filament-based process, Actin filament organization, Actin binding, Actin filament binding, Actin cytoskeleton, Actin filament bundle, Actin cytoskeleton, Actin filament bundle, Actin-binding, Nervous system, Central nervous system, Brain | actin, neural |

| | | | |
|---|---|---|---|
| ARPC5L | -1.76 | Actin cytoskeleton organization, Actin filament-based process, Actin filament organization, Regulation of actin filament-based process, Regulation of actin filament organization, Regulation of actin cytoskeleton organization, Regulation of actin polymerization or depolymerization, Regulation of actin filament polymerization, Actin binding, Actin filament binding, Actin cytoskeleton, Mixed, incl. actin filament organization, and filamentous actin, Mixed, incl. regulation of actin polymerization or depolymerization, and Calponin homology (CH) domain, RHO GTPases Activate WASPs and WAVEs, and actin filament organization, Actin cytoskeleton, Actin-binding, Synapse, Glutamatergic synapse | actin, neural |
| TJP1 | -1.96 | Actin cytoskeleton organization, Actin filament-based process, Regulation of actin filament-based process, Regulation of actin filament organization, Regulation of actin cytoskeleton organization, Actin cytoskeleton, Actin cytoskeleton, Nervous system, Central nervous system, Brain | actin, neural |
| ENAH | -2.27 | Actin cytoskeleton organization, Actin filament-based process, Actin filament organization, Actin polymerization or depolymerization, Actin binding, Mixed, incl. actin filament organization, and filamentous actin, Mixed, incl. regulation of actin polymerization or depolymerization, and Calponin homology (CH) domain, RHO GTPases Activate WASPs and WAVEs, and actin filament organization, Actin-binding, Filopodium, Axon guidance, Synapse, Nervous system development | actin, neural |
| RDX | -2.59 | Regulation of actin filament-based process, Regulation of actin filament organization, Regulation of actin cytoskeleton organization, Regulation of actin filament depolymerization, Regulation of actin polymerization or depolymerization, Barbed-end actin filament capping, Negative regulation of actin filament depolymerization, Negative regulation of actin filament polymerization, Regulation of actin filament polymerization, Actin binding, Actin cytoskeleton, Cortical actin cytoskeleton, Actin cytoskeleton, Actin-binding, Filopodium, Filopodium, Axon guidance, Neuron projection, Nervous system development, Nervous system, Central nervous system, Brain, Forebrain, Cerebral cortex | actin, neural |
| TMSB10 | -3.59 | Actin cytoskeleton organization, Actin filament-based process, Actin filament organization, Regulation of actin filament-based process, Regulation of actin filament organization, Regulation of actin cytoskeleton organization, Regulation of actin polymerization or depolymerization, Negative regulation of actin filament polymerization, Regulation of actin filament polymerization, Actin binding, Actin-binding, Nervous system, Central nervous system, Brain | actin, neural |
| SDCBP | -3.61 | Actin cytoskeleton organization, Actin filament-based process, Axon guidance, Synapse, Nervous system development, Nervous system, Central nervous system, Brain | actin, neural |

| | | | |
|---|---|---|---|
| TMSB4X | -6.28 | Actin cytoskeleton organization, Actin filament-based process, Actin filament organization, Regulation of actin filament-based process, Regulation of actin filament organization, Regulation of actin cytoskeleton organization, Regulation of actin polymerization or depolymerization, Negative regulation of actin filament polymerization, Regulation of actin filament polymerization, Actin binding, Mixed, incl. actin filament organization, and filamentous actin, Mixed, incl. regulation of actin polymerization or depolymerization, and Calponin homology (CH) domain, RHO GTPases Activate WASPs and WAVEs, and actin filament organization, Actin-binding, Nervous system, Central nervous system, Brain | actin, neural |
| CD63 | 4.7 | Nervous system, Central nervous system, Brain | neural |
| PRNP | 4.38 | Axon, Distal axon, Axon guidance, Neuron projection, Somatodendritic compartment, Dendrite, Postsynapse organization, Synapse, Postsynaptic density, Postsynapse, Nervous system development, Nervous system, Central nervous system, Brain, Forebrain, Cerebral cortex | neural |
| MCFD2 | 4.33 | Nervous system, Central nervous system, Brain | neural |
| FAF2 | 4.17 | Nervous system, Central nervous system, Brain | neural |
| AKR7A2 | 3.77 | Nervous system, Central nervous system, Brain | neural |
| AGFG1 | 3.71 | Neuronal cell body, Somatodendritic compartment | neural |
| HMGA1 | 3.69 | Nervous system, Central nervous system, Brain | neural |
| LMO7 | 3.59 | Nervous system, Central nervous system, Brain | neural |
| SOD1 | 3.56 | Axon, Neuron projection, Neuronal cell body, Somatodendritic compartment, Dendrite, Nervous system, Central nervous system, Brain, Brain cell line, Forebrain, Cerebral cortex | neural |
| GRPEL1 | 3.51 | Nervous system, Central nervous system, Brain | neural |
| CDH2 | 3.41 | Neuron projection, Postsynapse organization, Synapse, Postsynaptic density, Postsynapse, Nervous system, Central nervous system, Brain | neural |
| COL1A1 | 3.28 | Nervous system, Central nervous system, Brain, Brain cell line | neural |
| SH3GLB1 | 3.2 | Nervous system, Central nervous system, Brain | neural |
| PSMD4 | 3.18 | Axon guidance, Nervous system development, Nervous system, Central nervous system, Brain, Wnt signaling pathway, planar cell polarity pathway | neural |
| GDI1 | 3.13 | Positive regulation of axon extension, Axon, Neuron projection, Neuronal cell body, Somatodendritic compartment, Nervous system, Central nervous system, Brain | neural |
| MAP4K4 | 3.08 | Nervous system, Central nervous system, Brain | neural |
| RAB1A | 3.08 | Nervous system, Central nervous system, Brain | neural |
| RAPH1 | 3.05 | Filopodium, Filopodium | neural |
| PDHB | 3.04 | Nervous system, Central nervous system, Brain, Forebrain, Cerebral cortex | neural |
| ACP2 | 3.03 | Nervous system, Central nervous system, Brain, Forebrain | neural |
| CALM2 | 3.01 | Nervous system, Central nervous system, Brain, Forebrain, Cerebral cortex, Basal ganglion, Ganglion | neural |

| | | | |
|---|---|---|---|
| GRAMD3 | 2.98 | Nervous system, Central nervous system, Brain | neural |
| ATP2B1 | 2.96 | Neuron projection, Neuronal cell body, Somatodendritic compartment, Synapse, Glutamatergic synapse, Nervous system, Central nervous system, Brain, Forebrain, Cerebral cortex | neural |
| UBQLN2 | 2.93 | Nervous system, Central nervous system, Brain, Forebrain, Cerebral cortex, Amygdala, Basal ganglion, Ganglion | neural |
| CERCAM | 2.89 | Nervous system, Central nervous system, Brain, Brain cell line | neural |
| AKAP12 | 2.88 | Neuronal cell body, Somatodendritic compartment, Synapse, Nervous system, Central nervous system, Brain | neural |
| TSNAX | 2.85 | Nervous system, Central nervous system, Brain, Forebrain, Cerebral cortex, Basal ganglion, Ganglion | neural |
| PRKRA | 2.81 | Nervous system, Central nervous system, Brain | neural |
| ENG | 2.79 | Nervous system, Central nervous system, Brain | neural |
| VAMP3 | 2.78 | Neuron projection, Synapse, Nervous system, Central nervous system, Brain | neural |
| AHNAK2 | 2.77 | Nervous system, Central nervous system, Brain, Forebrain, Cerebral cortex | neural |
| IFITM3 | 2.72 | Nervous system, Central nervous system, Brain | neural |
| DYNLT1 | 2.71 | Growth cone, Axon, Distal axon, Neuron projection, Neuronal cell body, Somatodendritic compartment, Nervous system, Central nervous system, Brain | neural |
| PAFAH1B2 | 2.71 | Nervous system, Central nervous system, Brain | neural |
| HN1 | 2.69 | Nervous system, Central nervous system, Brain | neural |
| ATP5H | 2.67 | Nervous system, Central nervous system, Brain | neural |
| ZMPSTE24 | 2.65 | Nervous system, Central nervous system, Brain | neural |
| CLTA | 2.64 | Axon guidance, Synapse, Postsynapse, Presynaptic endocytic zone membrane, Postsynaptic endocytic zone cytoplasmic component, Nervous system development, Nervous system, Central nervous system, Brain, Forebrain, Cerebral cortex | neural |
| PICALM | 2.64 | Neuronal cell body, Somatodendritic compartment, Synapse, Postsynapse, Presynaptic endocytic zone membrane, Nervous system, Central nervous system, Brain | neural |
| CACYBP | 2.6 | Neuron projection, Nervous system, Central nervous system, Brain | neural |
| SLC30A1 | 2.6 | Synapse, Postsynaptic density, Postsynapse | neural |
| TPD52L2 | 2.6 | Nervous system, Central nervous system, Brain | neural |
| BAG2 | 2.58 | Axon, Neuron projection, Somatodendritic compartment, Dendrite, Nervous system | neural |
| GCC1 | 2.56 | Nervous system, Central nervous system | neural |
| ITGA2 | 2.55 | Axon, Distal axon, Axon guidance, Neuron projection, Synapse, Nervous system development | neural |
| HERC4 | 2.53 | Nervous system, Central nervous system, Brain | neural |
| CTBP1 | 2.5 | Synapse, Glutamatergic synapse, Nervous system, Central nervous system, Brain | neural |
| HSPB1 | 2.5 | Axon, Neuron projection, Nervous system, Central nervous system, Brain | neural |
| ETFA | 2.47 | Brain cell line | neural |

| | | | |
|---|---|---|---|
| STAT3 | 2.44 | Synapse, Postsynaptic density, Postsynapse, Glutamatergic synapse, Nervous system, Central nervous system, Brain | neural |
| TOLLIP | 2.44 | Nervous system, Central nervous system, Brain | neural |
| PSMD5 | 2.42 | Axon guidance, Nervous system development, Wnt signaling pathway, planar cell polarity pathway | neural |
| SCYL1 | 2.42 | Nervous system, Central nervous system, Brain, Forebrain | neural |
| BOLA2 | 2.4 | Nervous system, Central nervous system, Brain | neural |
| HADHA | 2.4 | Nervous system, Central nervous system, Brain, Forebrain, Cerebral cortex | neural |
| STX4 | 2.4 | Neuron projection, Somatodendritic compartment, Dendrite, Synapse, Postsynapse, Glutamatergic synapse | neural |
| PRRC1 | 2.39 | Nervous system, Central nervous system | neural |
| ZNF207 | 2.37 | Nervous system, Central nervous system, Brain | neural |
| FKBP1A | 2.35 | Nervous system, Central nervous system, Brain | neural |
| KIAA1715 | 2.34 | Nervous system, Central nervous system, Brain | neural |
| CTNND1 | 2.33 | Growth cone, Axon, Distal axon, Neuron projection, Somatodendritic compartment, Dendrite, Synapse, Postsynaptic density, Postsynapse, Glutamatergic synapse, Nervous system, Central nervous system, Brain | neural |
| HSPE1 | 2.33 | Nervous system, Central nervous system, Brain | neural |
| SPAG9 | 2.33 | Nervous system, Central nervous system, Brain | neural |
| CMPK1 | 2.32 | Nervous system, Central nervous system, Brain, Forebrain | neural |
| AP2A1 | 2.31 | Filopodium, Axon guidance, Nervous system development, Wnt signaling pathway, planar cell polarity pathway | neural |
| FTL | 2.31 | Nervous system, Central nervous system, Brain | neural |
| BANF1 | 2.3 | Nervous system, Central nervous system, Brain | neural |
| SLC12A4 | 2.28 | Synapse, Nervous system, Central nervous system, Brain | neural |
| RAB5C | 2.27 | Nervous system, Central nervous system, Brain | neural |
| C1orf123 | 2.25 | Nervous system, Central nervous system, Brain | neural |
| TBCD | 2.24 | Nervous system, Central nervous system, Brain | neural |
| AP2M1 | 2.23 | Axon guidance, Nervous system development, Nervous system, Central nervous system, Brain, Wnt signaling pathway, planar cell polarity pathway | neural |
| GSS | 2.23 | Nervous system, Central nervous system, Brain, Forebrain, Cerebral cortex, Basal ganglion, Ganglion | neural |
| COPS4 | 2.21 | Synapse, Nervous system, Central nervous system, Brain | neural |
| RIC8A | 2.21 | Nervous system, Central nervous system, Brain | neural |
| PDXDC1 | 2.2 | Nervous system, Central nervous system, Brain | neural |
| PHLDB1 | 2.2 | Nervous system, Central nervous system, Brain | neural |
| UBE2Z | 2.2 | Nervous system, Central nervous system, Brain | neural |
| NUDC | 2.19 | Nervous system, Central nervous system, Brain | neural |
| TGM2 | 2.18 | Nervous system, Central nervous system, Brain | neural |
| YKT6 | 2.16 | Neuron projection, Neuronal cell body, Somatodendritic compartment, Dendrite, Nervous system | neural |
| AK4 | 2.11 | Nervous system, Central nervous system, Brain | neural |
| ARFGAP2 | 2.09 | Nervous system, Central nervous system, Brain | neural |

| | | | |
|---|---|---|---|
| EEF1B2 | 2.09 | Nervous system, Central nervous system, Brain | neural |
| COPS3 | 2.08 | Nervous system, Central nervous system, Brain, Forebrain, Cerebral cortex | neural |
| MAVS | 2.08 | Nervous system, Central nervous system, Brain | neural |
| SEC24A | 2.08 | Nervous system, Central nervous system, Brain | neural |
| GLS | 2.07 | Synapse, Nervous system, Central nervous system, Brain | neural |
| ECH1 | 2.06 | Nervous system, Central nervous system, Brain | neural |
| IPO5 | 2.04 | Nervous system, Central nervous system, Brain | neural |
| RAB31 | 2.04 | Nervous system, Central nervous system, Brain | neural |
| PURA | 2.03 | Neuron projection, Neuronal cell body, Somatodendritic compartment, Dendrite, Synapse, Postsynapse, Glutamatergic synapse | neural |
| EHD1 | 2 | Nervous system, Central nervous system, Brain | neural |
| EXOC2 | 2 | Nervous system, Central nervous system, Brain | neural |
| YAP1 | 1.99 | Nervous system development, Nervous system, Central nervous system, Brain, Forebrain | neural |
| TMPO | 1.98 | Nervous system, Central nervous system, Brain | neural |
| SNAP23 | 1.95 | Neuron projection, Synapse | neural |
| CYBRD1 | 1.94 | Nervous system, Central nervous system, Brain | neural |
| SCAMP1 | 1.94 | Synapse | neural |
| C14orf166 | 1.93 | Nervous system, Central nervous system, Brain | neural |
| TMEM214 | 1.93 | Nervous system, Central nervous system, Brain, Forebrain, Cerebral cortex, Amygdala, Basal ganglion, Ganglion | neural |
| PPME1 | 1.89 | Nervous system, Central nervous system, Brain, Forebrain, Cerebral cortex, Basal ganglion, Ganglion | neural |
| CD81 | 1.88 | Nervous system, Central nervous system, Brain | neural |
| ALCAM | 1.87 | Axon, Axon guidance, Neuron projection, Neuronal cell body, Somatodendritic compartment, Dendrite, Nervous system development, Nervous system, Central nervous system, Brain | neural |
| CARS | 1.85 | Nervous system, Central nervous system, Brain | neural |
| PRPSAP1 | 1.85 | Nervous system, Central nervous system, Brain | neural |
| STRN | 1.84 | Neuron projection, Neuronal cell body, Somatodendritic compartment, Dendrite, Synapse, Postsynaptic density, Postsynapse | neural |
| IMMT | 1.83 | Nervous system, Central nervous system, Brain, Brain cell line | neural |
| M6PR | 1.83 | Nervous system, Central nervous system, Brain | neural |
| GSK3B | 1.82 | Axon, Axon guidance, Neuron projection, Somatodendritic compartment, Dendrite, Synapse, Postsynapse, Glutamatergic synapse, Nervous system development | neural |
| UNC45A | 1.82 | Nervous system, Central nervous system, Brain | neural |
| WDR44 | 1.78 | Nervous system, Central nervous system, Brain | neural |
| GALK1 | 1.77 | Nervous system, Central nervous system, Brain | neural |
| ATP6V1C1 | 1.76 | Nervous system, Central nervous system, Brain | neural |
| EEF1D | 1.75 | Nervous system, Central nervous system, Brain | neural |
| ERGIC1 | 1.75 | Nervous system, Central nervous system, Brain, Forebrain | neural |
| CCDC80 | 1.74 | Nervous system, Central nervous system, Brain | neural |

| | | | |
|---|---|---|---|
| PAICS | 1.73 | Nervous system, Central nervous system, Brain | neural |
| PEA15 | 1.73 | Nervous system, Central nervous system, Brain, Brain cell line | neural |
| AAK1 | 1.72 | Axon, Distal axon, Neuron projection, Synapse, Nervous system, Central nervous system, Brain | neural |
| SPATS2L | 1.72 | Nervous system, Central nervous system, Brain, Ganglion | neural |
| PDIA6 | 1.71 | Nervous system, Central nervous system, Brain, Forebrain, Cerebral cortex, Amygdala, Basal ganglion, Ganglion | neural |
| ASAP2 | 1.7 | Nervous system, Central nervous system, Brain | neural |
| SEC16A | 1.7 | Nervous system, Central nervous system, Brain | neural |
| UQCRC1 | 1.69 | Nervous system, Central nervous system, Brain, Forebrain, Cerebral cortex, Basal ganglion, Ganglion | neural |
| TUBB4A | 1.68 | Axon, Axon guidance, Neuron projection, Neuronal cell body, Somatodendritic compartment, Nervous system development, Nervous system, Central nervous system, Brain | neural |
| NEU1 | 1.66 | Nervous system, Central nervous system, Brain | neural |
| STXBP1 | 1.63 | Axon, Neuron projection, Synapse, Postsynapse, Glutamatergic synapse, Nervous system, Central nervous system, Brain, Forebrain, Cerebral cortex | neural |
| BSG | 1.62 | Axon, Neuron projection, Nervous system, Central nervous system, Brain | neural |
| CLTB | 1.62 | Axon guidance, Synapse, Postsynapse, Presynaptic endocytic zone membrane, Postsynaptic endocytic zone cytoplasmic component, Nervous system development | neural |
| PGM1 | 1.62 | Nervous system, Central nervous system, Brain, Forebrain | neural |
| IFI16 | 1.61 | Nervous system, Central nervous system, Brain, Forebrain | neural |
| PPM1G | 1.61 | Nervous system, Central nervous system, Brain | neural |
| STAT1 | 1.61 | Axon, Neuron projection, Somatodendritic compartment, Dendrite, Nervous system, Central nervous system, Brain | neural |
| UBA1 | 1.61 | Nervous system, Central nervous system, Brain | neural |
| THY1 | 1.6 | Growth cone, Axon, Distal axon, Neuron projection, Neuronal cell body, Somatodendritic compartment, Dendrite, Nervous system, Central nervous system, Brain, Forebrain, Cerebral cortex, Amygdala, Basal ganglion, Ganglion | neural |
| CCT2 | 1.59 | Nervous system, Central nervous system, Brain, Brain cell line, Forebrain, Cerebral cortex, Amygdala, Basal ganglion, Ganglion | neural |
| LAMP2 | 1.59 | Nervous system, Central nervous system, Brain | neural |
| DNAJB1 | 1.57 | Neuron projection, Neuronal cell body, Somatodendritic compartment, Dendrite, Synapse, Postsynaptic density, Postsynapse, Glutamatergic synapse, Nervous system, Central nervous system, Brain | neural |
| CCT6A | 1.56 | Nervous system, Central nervous system, Brain | neural |
| NDRG1 | 1.56 | Postsynapse organization, Synapse, Glutamatergic synapse, Nervous system, Central nervous system, Brain, Brain cell line | neural |

| | | | |
|---|---|---|---|
| PPP3CA | 1.56 | Neuron projection, Somatodendritic compartment, Dendrite, Synapse, Postsynapse, Glutamatergic synapse, Nervous system, Central nervous system, Brain, Forebrain, Cerebral cortex, Amygdala, Basal ganglion, Ganglion | neural |
| COL5A2 | 1.54 | Axon guidance, Nervous system development, Nervous system, Central nervous system, Brain | neural |
| ARL6IP5 | 1.53 | Nervous system, Central nervous system, Brain | neural |
| SEMA7A | 1.51 | Positive regulation of axon extension, Axon guidance, Nervous system development, Nervous system, Central nervous system, Brain, Forebrain, Cerebral cortex | neural |
| TUBB3 | 1.51 | Filopodium, Filopodium, Growth cone, Axon, Distal axon, Axon guidance, Neuron projection, Somatodendritic compartment, Dendrite, Nervous system development, Brain cell line | neural |
| HIST1H3A | -1.55 | Nervous system, Central nervous system, Brain, Forebrain, Cerebral cortex, Basal ganglion, Ganglion | neural |
| PSPH | -1.55 | Neuron projection | neural |
| ASMTL | -1.57 | Nervous system, Central nervous system, Brain | neural |
| CCAR1 | -1.57 | Nervous system, Central nervous system, Brain | neural |
| HSD17B12 | -1.59 | Nervous system, Central nervous system, Brain | neural |
| PSMB5 | -1.63 | Axon guidance, Nervous system development, Nervous system, Central nervous system, Brain, Forebrain, Wnt signaling pathway, planar cell polarity pathway | neural |
| RRAGD | -1.69 | Nervous system, Central nervous system, Brain | neural |
| TUBB8 | -1.72 | Axon guidance, Nervous system development | neural |
| SERBP1 | -1.77 | Nervous system, Central nervous system, Brain | neural |
| UQCRFS1 | -1.79 | Nervous system, Central nervous system, Brain | neural |
| PSAT1 | -1.8 | Nervous system, Central nervous system, Brain | neural |
| TMED10 | -1.8 | Nervous system, Central nervous system, Brain, Forebrain, Cerebral cortex, Amygdala, Basal ganglion, Ganglion | neural |
| HIST1H4A | -1.81 | Nervous system, Central nervous system, Brain | neural |
| BLMH | -1.86 | Nervous system, Central nervous system, Brain | neural |
| COL6A3 | -1.86 | Axon guidance, Nervous system development | neural |
| BASP1 | -1.87 | Growth cone, Axon, Distal axon, Neuron projection, Nervous system, Central nervous system, Brain, Forebrain, Cerebral cortex, Basal ganglion, Ganglion | neural |
| FKBP9 | -1.87 | Nervous system | neural |
| NQO1 | -1.89 | Neuron projection, Neuronal cell body, Somatodendritic compartment, Dendrite, Synapse, Nervous system, Central nervous system, Brain, Forebrain, Cerebral cortex, Basal ganglion, Ganglion | neural |
| SRSF3 | -1.89 | Nervous system, Central nervous system, Brain | neural |
| TMED4 | -1.93 | Nervous system, Central nervous system, Brain | neural |
| FKBP7 | -1.94 | Nervous system, Central nervous system, Brain | neural |
| LMNB1 | -1.94 | Nervous system, Central nervous system, Brain, Brain cell line | neural |
| CDH13 | -1.99 | Neuron projection, Synapse, Nervous system, Central nervous system, Brain | neural |

| | | | |
|---|---|---|---|
| YWHAE | -2.03 | Growth cone, Axon, Distal axon, Neuron projection, Synapse, Glutamatergic synapse, Nervous system, Central nervous system, Brain, Forebrain, Cerebral cortex, Basal ganglion, Ganglion | neural |
| BZW1 | -2.15 | Nervous system, Central nervous system, Brain | neural |
| PSMA7 | -2.16 | Axon guidance, Synapse, Postsynapse, Nervous system development, Nervous system, Central nervous system, Brain, Wnt signaling pathway, planar cell polarity pathway | neural |
| UBLCP1 | -2.2 | Nervous system, Central nervous system, Brain | neural |
| SLC16A3 | -2.22 | Synapse, Postsynaptic density, Postsynapse, Nervous system, Central nervous system, Brain, Forebrain, Cerebral cortex | neural |
| ACSL3 | -2.27 | Nervous system, Central nervous system, Brain | neural |
| UBE2K | -2.28 | Filopodium, Nervous system, Central nervous system, Brain | neural |
| CRYZ | -2.29 | Nervous system, Central nervous system, Brain, Forebrain | neural |
| ILVBL | -2.4 | Nervous system, Central nervous system, Brain | neural |
| PSMB6 | -2.49 | Axon guidance, Nervous system development, Nervous system, Central nervous system, Brain, Wnt signaling pathway, planar cell polarity pathway | neural |
| HSDL2 | -2.54 | Nervous system, Central nervous system, Brain | neural |
| MGST3 | -2.54 | Nervous system, Central nervous system, Brain | neural |
| NQO2 | -2.54 | Nervous system, Central nervous system, Brain | neural |
| PPT1 | -2.59 | Axon, Neuron projection, Neuronal cell body, Somatodendritic compartment, Dendrite, Synapse, Nervous system, Central nervous system, Brain | neural |
| SSR2 | -2.65 | Nervous system, Central nervous system, Brain | neural |
| RPA1 | -2.71 | Nervous system, Central nervous system, Brain | neural |
| UBE2I | -2.71 | Nervous system, Central nervous system, Brain | neural |
| NIPSNAP3A | -2.82 | Nervous system, Central nervous system, Brain | neural |
| LGMN | -2.83 | Postsynapse organization | neural |
| NACA | -2.83 | Nervous system, Central nervous system, Brain | neural |
| MTHFD2 | -2.87 | Nervous system, Central nervous system, Brain | neural |
| EWSR1 | -2.98 | Nervous system, Central nervous system, Brain | neural |
| GAP43 | -3.08 | Filopodium, Filopodium, Growth cone, Axon, Distal axon, Axon guidance, Neuron projection, Neuronal cell body, Somatodendritic compartment, Dendrite, Synapse, Postsynaptic density, Postsynapse, Nervous system development, Nervous system, Central nervous system, Brain, Forebrain, Cerebral cortex, Basal ganglion, Ganglion | neural |
| PSMA3 | -3.1 | Axon guidance, Synapse, Nervous system development, Wnt signaling pathway, planar cell polarity pathway | neural |
| GSTM3 | -3.12 | Nervous system, Central nervous system, Brain | neural |
| H2AFV | -3.26 | Nervous system, Central nervous system, Brain | neural |
| ACAT2 | -3.52 | Nervous system, Central nervous system, Brain, Forebrain, Cerebral cortex, Amygdala, Basal ganglion, Ganglion | neural |
| XXYLT1 | -3.71 | Nervous system | neural |
| B2M | -3.87 | Nervous system, Central nervous system, Brain | neural |
| ATP5D | -3.92 | Nervous system, Central nervous system, Brain | neural |

| | | | |
|---|---|---|---|
| ARF5 | -4.04 | Nervous system, Central nervous system, Brain | neural |
| MAP1LC3B | -4.29 | Nervous system, Central nervous system, Brain | neural |
| RBBP4 | -4.56 | Nervous system, Central nervous system, Brain | neural |
| RSU1 | -5.15 | Nervous system, Central nervous system, Brain, Brain cell line, Forebrain | neural |
| UBE2D3 | -5.8 | Nervous system, Central nervous system, Brain | neural |
| EIF4H | 3.32 | Nervous system, Central nervous system, Brain, Ribonucleoprotein complex, GTP hydrolysis and joining of the 60S ribosomal subunit, Ribosomal scanning and start codon recognition | neural, ribosome |
| CARHSP1 | 3.28 | Nervous system, Central nervous system, Brain, Cytoplasmic ribonucleoprotein granule | neural, ribosome |
| KLC1 | 3.21 | Growth cone, Axon, Distal axon, Neuron projection, Nervous system, Central nervous system, Brain, Ribonucleoprotein complex subunit organization | neural, ribosome |
| EIF3E | 3.15 | Nervous system, Central nervous system, Brain, Ribonucleoprotein complex biogenesis, Ribonucleoprotein complex subunit organization, Ribonucleoprotein complex assembly, Ribonucleoprotein complex, GTP hydrolysis and joining of the 60S ribosomal subunit, Ribosomal scanning and start codon recognition, Ribonucleoprotein complex | neural, ribosome |
| KHSRP | 3.09 | Nervous system, Central nervous system, Brain, Brain cell line, Cytoplasmic ribonucleoprotein granule | neural, ribosome |
| SQSTM1 | 2.79 | Nervous system, Central nervous system, Brain, Forebrain, Cerebral cortex, Basal ganglion, Ganglion, Cytoplasmic ribonucleoprotein granule | neural, ribosome |
| PCBP1 | 2.64 | Nervous system, Central nervous system, Brain, Cytoplasmic ribonucleoprotein granule, Ribonucleoprotein | neural, ribosome |
| RPL24 | 2.58 | Axon guidance, Nervous system development, Ribosome assembly, Ribonucleoprotein complex biogenesis, Ribonucleoprotein complex subunit organization, Ribonucleoprotein complex assembly, Ribosome biogenesis, Structural constituent of ribosome, Cytosolic ribosome, Ribonucleoprotein complex, Ribosome, Ribosomal subunit, Cytosolic large ribosomal subunit, Large ribosomal subunit, Cytoplasmic ribosomal proteins, Cytoplasmic ribosomal proteins, Cytoplasmic ribosomal proteins, Ribosome, GTP hydrolysis and joining of the 60S ribosomal subunit, Cytoplasmic ribosomal proteins, Cytosolic ribosome, Ribonucleoprotein complex, Ribosome, Ribosomal subunit, Cytosolic large ribosomal subunit, Large ribosomal subunit, Ribonucleoprotein, Ribosomal protein | neural, ribosome |
| UBAP2L | 2.21 | Nervous system, Central nervous system, Brain, Cytoplasmic ribonucleoprotein granule | neural, ribosome |

| | | | |
|---|---|---|---|
| RPS10 | 2.18 | Axon guidance, Nervous system development, Nervous system, Central nervous system, Brain, Ribosome assembly, Ribonucleoprotein complex biogenesis, Ribonucleoprotein complex subunit organization, Ribonucleoprotein complex assembly, Ribosome biogenesis, Structural constituent of ribosome, Cytosolic ribosome, Ribonucleoprotein complex, Ribosome, Ribosomal subunit, Cytosolic small ribosomal subunit, Cytoplasmic ribosomal proteins, Cytoplasmic ribosomal proteins, Cytoplasmic ribosomal proteins, Ribosome, GTP hydrolysis and joining of the 60S ribosomal subunit, Ribosomal scanning and start codon recognition, Cytoplasmic ribosomal proteins, Cytosolic ribosome, Ribonucleoprotein complex, Ribosome, Ribosomal subunit, Cytosolic small ribosomal subunit, Ribonucleoprotein, Ribosomal protein | neural, ribosome |
| API5 | 2.17 | Nervous system, Central nervous system, Brain, Ribonucleoprotein complex, Ribonucleoprotein complex | neural, ribosome |
| CIRBP | 2.14 | Nervous system, Central nervous system, Brain, Ribonucleoprotein complex, Cytoplasmic ribonucleoprotein granule | neural, ribosome |
| HSD17B10 | 2.14 | Nervous system, Central nervous system, Brain, Ribonucleoprotein complex, Ribonucleoprotein complex | neural, ribosome |
| RBM3 | 2.14 | Neuron projection, Somatodendritic compartment, Dendrite, Nervous system, Central nervous system, Brain, Ribonucleoprotein complex binding, Ribonucleoprotein complex | neural, ribosome |
| CUL4B | 2.1 | Nervous system, Central nervous system, Brain, Ribonucleoprotein complex biogenesis, Ribosome biogenesis | neural, ribosome |
| MRPL50 | 1.93 | Nervous system, Central nervous system, Brain, Forebrain, Cerebral cortex, Ribonucleoprotein complex, Ribosome, Ribosomal subunit, Large ribosomal subunit, Ribonucleoprotein complex, Ribosome, Ribosomal subunit, Large ribosomal subunit, Ribonucleoprotein, Ribosomal protein | neural, ribosome |
| EIF3F | 1.9 | Nervous system, Central nervous system, Brain, Forebrain, Cerebral cortex, Amygdala, Basal ganglion, Ganglion, Ribonucleoprotein complex biogenesis, Ribonucleoprotein complex subunit organization, Ribonucleoprotein complex assembly, Ribonucleoprotein complex, GTP hydrolysis and joining of the 60S ribosomal subunit, Ribosomal scanning and start codon recognition, Ribonucleoprotein complex | neural, ribosome |
| FXR2 | 1.88 | Filopodium, Filopodium, Growth cone, Axon, Distal axon, Neuron projection, Neuronal cell body, Somatodendritic compartment, Dendritic spine neck, Dendrite, Synapse, Postsynaptic density, Postsynapse, Nervous system, Central nervous system, Brain, Cytosolic ribosome, Ribonucleoprotein complex, Ribosome, Ribosomal subunit, Cytosolic large ribosomal subunit, Cytoplasmic ribonucleoprotein granule, Large ribosomal subunit, Cytosolic ribosome, Ribonucleoprotein complex, Ribosome, Ribosomal subunit, Cytosolic large ribosomal subunit, Large ribosomal subunit | neural, ribosome |

| | | | |
|---|---|---|---|
| EIF3M | 1.73 | Nervous system, Central nervous system, Brain, Forebrain, Ribonucleoprotein complex biogenesis, Ribonucleoprotein complex subunit organization, Ribonucleoprotein complex assembly, Ribonucleoprotein complex, GTP hydrolysis and joining of the 60S ribosomal subunit, Ribosomal scanning and start codon recognition | neural, ribosome |
| PNN | 1.7 | Nervous system, Central nervous system, Brain, Ribonucleoprotein complex, Ribonucleoprotein complex | neural, ribosome |
| NPM1 | 1.67 | Nervous system, Central nervous system, Brain, Ribosome assembly, Ribonucleoprotein complex biogenesis, Ribosome biogenesis, Ribonucleoprotein complex binding, Ribonucleoprotein complex, Ribonucleoprotein complex | neural, ribosome |
| CKAP5 | 1.65 | Nervous system, Ribonucleoprotein complex binding | neural, ribosome |
| HNRNPM | 1.59 | Nervous system, Central nervous system, Brain, Ribonucleoprotein complex, Ribonucleoprotein complex, Ribonucleoprotein | neural, ribosome |
| DDX19A | 1.56 | Nervous system, Central nervous system, Brain, Cytoplasmic ribonucleoprotein granule | neural, ribosome |
| FXR1 | -1.72 | Filopodium, Filopodium, Growth cone, Axon, Distal axon, Neuron projection, Neuronal cell body, Somatodendritic compartment, Dendritic spine neck, Dendrite, Synapse, Postsynaptic density, Postsynapse, Glutamatergic synapse, Nervous system, Central nervous system, Brain, Ribonucleoprotein complex, Cytoplasmic ribonucleoprotein granule, Ribonucleoprotein complex | neural, ribosome |
| RPS4X | -1.76 | Axon guidance, Synapse, Nervous system development, Nervous system, Central nervous system, Brain, Structural constituent of ribosome, Cytosolic ribosome, Ribonucleoprotein complex, Ribosome, Ribosomal subunit, Cytosolic small ribosomal subunit, Cytoplasmic ribonucleoprotein granule, Cytoplasmic ribosomal proteins, Cytoplasmic ribosomal proteins, Cytoplasmic ribosomal proteins, Ribosome, GTP hydrolysis and joining of the 60S ribosomal subunit, Ribosomal scanning and start codon recognition, Cytoplasmic ribosomal proteins, Cytosolic ribosome, Ribonucleoprotein complex, Ribosome, Ribosomal subunit, Cytosolic small ribosomal subunit, Ribonucleoprotein, Ribosomal protein | neural, ribosome |

| | | | |
|---|---|---|---|
| RPL23A | -1.8 | Axon guidance, Nervous system development, Nervous system, Central nervous system, Brain, Ribosome assembly, Ribonucleoprotein complex biogenesis, Ribonucleoprotein complex subunit organization, Ribonucleoprotein complex assembly, Ribosome biogenesis, Structural constituent of ribosome, Cytosolic ribosome, Ribonucleoprotein complex, Ribosome, Ribosomal subunit, Cytosolic large ribosomal subunit, Large ribosomal subunit, Cytoplasmic ribosomal proteins, Cytoplasmic ribosomal proteins, Cytoplasmic ribosomal proteins, Ribosome, GTP hydrolysis and joining of the 60S ribosomal subunit, Cytoplasmic ribosomal proteins, Cytosolic ribosome, Ribonucleoprotein complex, Ribosome, Ribosomal subunit, Cytosolic large ribosomal subunit, Large ribosomal subunit, Ribonucleoprotein, Ribosomal protein | neural, ribosome |
| PRMT5 | -1.81 | Nervous system, Central nervous system, Brain, Ribonucleoprotein complex biogenesis, Ribonucleoprotein complex subunit organization, Ribonucleoprotein complex assembly, Ribonucleoprotein complex binding | neural, ribosome |
| EIF4B | -1.84 | Nervous system, Central nervous system, Brain, Ribonucleoprotein complex, GTP hydrolysis and joining of the 60S ribosomal subunit, Ribosomal scanning and start codon recognition, Ribonucleoprotein complex | neural, ribosome |
| RPS5 | -1.89 | Axon guidance, Nervous system development, Nervous system, Central nervous system, Brain, Ribosome assembly, Ribonucleoprotein complex biogenesis, Ribonucleoprotein complex subunit organization, Ribonucleoprotein complex assembly, Ribosome biogenesis, Structural constituent of ribosome, Cytosolic ribosome, Ribonucleoprotein complex, Ribosome, Ribosomal subunit, Cytosolic small ribosomal subunit, Cytoplasmic ribosomal proteins, Cytoplasmic ribosomal proteins, Cytoplasmic ribosomal proteins, Ribosome, GTP hydrolysis and joining of the 60S ribosomal subunit, Ribosomal scanning and start codon recognition, Cytoplasmic ribosomal proteins, Cytosolic ribosome, Ribonucleoprotein complex, Ribosome, Ribosomal subunit, Cytosolic small ribosomal subunit, Ribonucleoprotein, Ribosomal protein | neural, ribosome |
| SRRT | -2.07 | Nervous system, Central nervous system, Brain, Forebrain, Ribonucleoprotein complex | neural, ribosome |
| SF1 | -2.09 | Nervous system, Central nervous system, Brain, Forebrain, Cerebral cortex, Ribonucleoprotein complex biogenesis, Ribonucleoprotein complex subunit organization, Ribonucleoprotein complex assembly, Ribonucleoprotein complex, Ribosome, Ribonucleoprotein complex, Ribosome | neural, ribosome |

| | | | |
|---|---|---|---|
| DHX29 | -2.1 | Nervous system, Central nervous system, Brain, Ribosome assembly, Ribonucleoprotein complex biogenesis, Ribonucleoprotein complex subunit organization, Ribonucleoprotein complex assembly, Ribosome biogenesis, Ribonucleoprotein complex binding, Cytosolic ribosome, Ribonucleoprotein complex, Ribosome, Ribosomal subunit, Cytosolic small ribosomal subunit, Cytosolic ribosome, Ribonucleoprotein complex, Ribosome, Ribosomal subunit, Cytosolic small ribosomal subunit | neural, ribosome |
| RSL1D1 | -2.12 | Nervous system, Central nervous system, Brain, Forebrain, Cerebral cortex, Basal ganglion, Ganglion, Ribonucleoprotein complex biogenesis, Ribosome biogenesis, Ribonucleoprotein complex, Ribonucleoprotein complex | neural, ribosome |
| RPL15 | -2.18 | Axon guidance, Nervous system development, Nervous system, Central nervous system, Brain, Structural constituent of ribosome, Cytosolic ribosome, Ribonucleoprotein complex, Ribosome, Ribosomal subunit, Cytosolic large ribosomal subunit, Large ribosomal subunit, Cytoplasmic ribosomal proteins, Cytoplasmic ribosomal proteins, Cytoplasmic ribosomal proteins, Ribosome, GTP hydrolysis and joining of the 60S ribosomal subunit, Cytoplasmic ribosomal proteins, Cytosolic ribosome, Ribonucleoprotein complex, Ribosome, Ribosomal subunit, Cytosolic large ribosomal subunit, Large ribosomal subunit, Ribonucleoprotein, Ribosomal protein | neural, ribosome |
| EIF1AX | -2.21 | Nervous system, Central nervous system, Brain, Forebrain, GTP hydrolysis and joining of the 60S ribosomal subunit, Ribosomal scanning and start codon recognition | neural, ribosome |
| MAGOHB | -2.31 | Axon guidance, Neuronal cell body, Somatodendritic compartment, Nervous system development, Nervous system, Central nervous system, Brain, Ribonucleoprotein complex, Ribonucleoprotein complex | neural, ribosome |
| RPS11 | -2.41 | Axon guidance, Nervous system development, Nervous system, Central nervous system, Brain, Structural constituent of ribosome, Cytosolic ribosome, Ribonucleoprotein complex, Ribosome, Ribosomal subunit, Cytosolic small ribosomal subunit, Cytoplasmic ribosomal proteins, Ribosome, GTP hydrolysis and joining of the 60S ribosomal subunit, Ribosomal scanning and start codon recognition, Cytoplasmic ribosomal proteins, Cytosolic ribosome, Ribonucleoprotein complex, Ribosome, Ribosomal subunit, Cytosolic small ribosomal subunit, Ribonucleoprotein, Ribosomal protein | neural, ribosome |

| | | | |
|---|---|---|---|
| RPS6 | -2.63 | Axon guidance, Neuron projection, Somatodendritic compartment, Dendrite, Nervous system development, Nervous system, Central nervous system, Brain, Ribonucleoprotein complex biogenesis, Ribosome biogenesis, Structural constituent of ribosome, Cytosolic ribosome, Ribonucleoprotein complex, Ribosome, Ribosomal subunit, Cytosolic small ribosomal subunit, Cytoplasmic ribonucleoprotein granule, Cytoplasmic ribosomal proteins, Ribosome, GTP hydrolysis and joining of the 60S ribosomal subunit, Ribosomal scanning and start codon recognition, Cytoplasmic ribosomal proteins, Cytosolic ribosome, Ribonucleoprotein complex, Ribosome, Ribosomal subunit, Cytosolic small ribosomal subunit, Ribonucleoprotein, Ribosomal protein | neural, ribosome |
| RPL35 | -2.79 | Axon guidance, Nervous system development, Nervous system, Central nervous system, Brain, Ribonucleoprotein complex biogenesis, Ribosome biogenesis, Structural constituent of ribosome, Cytosolic ribosome, Ribonucleoprotein complex, Ribosome, Ribosomal subunit, Cytosolic large ribosomal subunit, Large ribosomal subunit, Cytoplasmic ribosomal proteins, Ribosome, GTP hydrolysis and joining of the 60S ribosomal subunit, Cytoplasmic ribosomal proteins, Cytosolic ribosome, Ribonucleoprotein complex, Ribosome, Ribosomal subunit, Cytosolic large ribosomal subunit, Large ribosomal subunit, Ribonucleoprotein, Ribosomal protein | neural, ribosome |
| PSMA2 | -2.87 | Axon guidance, Nervous system development, Nervous system, Central nervous system, Brain, Wnt signaling pathway, planar cell polarity pathway, Cytoplasmic ribonucleoprotein granule | neural, ribosome |
| NOP56 | -2.99 | Nervous system, Central nervous system, Brain, Ribonucleoprotein complex biogenesis, Ribosome biogenesis, Ribonucleoprotein complex, Ribonucleoprotein complex, Ribonucleoprotein | neural, ribosome |
| SEC61A1 | -3.03 | Nervous system, Central nervous system, Brain, Ribonucleoprotein complex binding, Ribonucleoprotein complex | neural, ribosome |
| RPL13A | -3.24 | Axon guidance, Nervous system development, Nervous system, Central nervous system, Brain, Structural constituent of ribosome, Cytosolic ribosome, Ribonucleoprotein complex, Ribosome, Ribosomal subunit, Cytosolic large ribosomal subunit, Large ribosomal subunit, Ribosome, GTP hydrolysis and joining of the 60S ribosomal subunit, Cytoplasmic ribosomal proteins, Cytosolic ribosome, Ribonucleoprotein complex, Ribosome, Ribosomal subunit, Cytosolic large ribosomal subunit, Large ribosomal subunit, Ribonucleoprotein, Ribosomal protein | neural, ribosome |

| | | | |
|---|---|---|---|
| RPL26 | -3.64 | Axon guidance, Nervous system development, Nervous system, Central nervous system, Brain, Ribonucleoprotein complex biogenesis, Ribosome biogenesis, Structural constituent of ribosome, Cytosolic ribosome, Ribonucleoprotein complex, Ribosome, Ribosomal subunit, Cytosolic large ribosomal subunit, Large ribosomal subunit, Cytoplasmic ribosomal proteins, Cytoplasmic ribosomal proteins, Cytoplasmic ribosomal proteins, Ribosome, GTP hydrolysis and joining of the 60S ribosomal subunit, Cytoplasmic ribosomal proteins, Cytosolic ribosome, Ribonucleoprotein complex, Ribosome, Ribosomal subunit, Cytosolic large ribosomal subunit, Large ribosomal subunit, Ribonucleoprotein, Ribosomal protein | neural, ribosome |
| RPS15 | -3.65 | Axon guidance, Nervous system development, Nervous system, Central nervous system, Brain, Ribosome assembly, Ribonucleoprotein complex biogenesis, Ribonucleoprotein complex subunit organization, Ribonucleoprotein complex assembly, Ribosome biogenesis, Structural constituent of ribosome, Cytosolic ribosome, Ribonucleoprotein complex, Ribosome, Ribosomal subunit, Cytosolic small ribosomal subunit, Cytoplasmic ribosomal proteins, Cytoplasmic ribosomal proteins, Cytoplasmic ribosomal proteins, Ribosome, GTP hydrolysis and joining of the 60S ribosomal subunit, Ribosomal scanning and start codon recognition, Cytoplasmic ribosomal proteins, Cytosolic ribosome, Ribonucleoprotein complex, Ribosome, Ribosomal subunit, Cytosolic small ribosomal subunit, Ribonucleoprotein, Ribosomal protein | neural, ribosome |
| RPLP1 | -4.02 | Axon guidance, Nervous system development, Nervous system, Central nervous system, Brain, Structural constituent of ribosome, Ribonucleoprotein complex binding, Cytosolic ribosome, Ribonucleoprotein complex, Ribosome, Ribosomal subunit, Cytosolic large ribosomal subunit, Large ribosomal subunit, Cytoplasmic ribosomal proteins, Cytoplasmic ribosomal proteins, Ribosome, GTP hydrolysis and joining of the 60S ribosomal subunit, Cytoplasmic ribosomal proteins, Cytosolic ribosome, Ribonucleoprotein complex, Ribosome, Ribosomal subunit, Cytosolic large ribosomal subunit, Large ribosomal subunit, Ribonucleoprotein, Ribosomal protein | neural, ribosome |
| EIF6 | -4.58 | Nervous system, Central nervous system, Brain, Ribosome assembly, Ribonucleoprotein complex biogenesis, Ribonucleoprotein complex subunit organization, Ribonucleoprotein complex assembly, Ribosome biogenesis, Ribonucleoprotein complex binding, Ribonucleoprotein complex, Ribonucleoprotein complex | neural, ribosome |
| BTF3 | -6.61 | Nervous system, Central nervous system, Brain, Ribonucleoprotein complex, Ribosome | neural, ribosome |
| ALYREF | 3.47 | Ribonucleoprotein complex, Ribonucleoprotein complex | ribosome |
| DDX6 | 2.99 | Ribonucleoprotein complex, Cytoplasmic ribonucleoprotein granule, Ribonucleoprotein complex | ribosome |
| SERPINB1 | 2.74 | Cytoplasmic ribonucleoprotein granule | ribosome |

| | | | |
|---|---|---|---|
| SRP72 | 2.42 | Ribonucleoprotein complex binding, Ribonucleoprotein complex, Ribonucleoprotein complex, Ribonucleoprotein | ribosome |
| CUL4A | 1.88 | Ribonucleoprotein complex biogenesis, Ribosome biogenesis | ribosome |
| PWP1 | -1.92 | Ribonucleoprotein complex biogenesis, Ribosome biogenesis | ribosome |
| DCPS | -1.94 | Cytoplasmic ribonucleoprotein granule | ribosome |
| SNRPD1 | -2.28 | Ribonucleoprotein complex biogenesis, Ribonucleoprotein complex subunit organization, Ribonucleoprotein complex assembly, Ribonucleoprotein complex binding, Ribonucleoprotein complex, Ribonucleoprotein complex, Ribonucleoprotein | ribosome |
| FAU | -2.43 | Structural constituent of ribosome, Cytosolic ribosome, Ribonucleoprotein complex, Ribosome, Ribosomal subunit, Cytosolic small ribosomal subunit, Cytoplasmic ribosomal proteins, Ribosome, Cytoplasmic ribosomal proteins, Cytosolic ribosome, Ribonucleoprotein complex, Ribosome, Ribosomal subunit, Cytosolic small ribosomal subunit | ribosome |
| SRP68 | -3.09 | Ribonucleoprotein complex binding, Ribonucleoprotein complex, Ribosome, Ribonucleoprotein complex, Ribosome, Ribonucleoprotein | ribosome |
| DDX21 | -3.14 | Ribonucleoprotein complex biogenesis, Ribosome biogenesis | ribosome |

String permalink with network functional analysis: https://version-11-5.string-db.org/cgi/network?networkId=bFY8QJcX4JxW

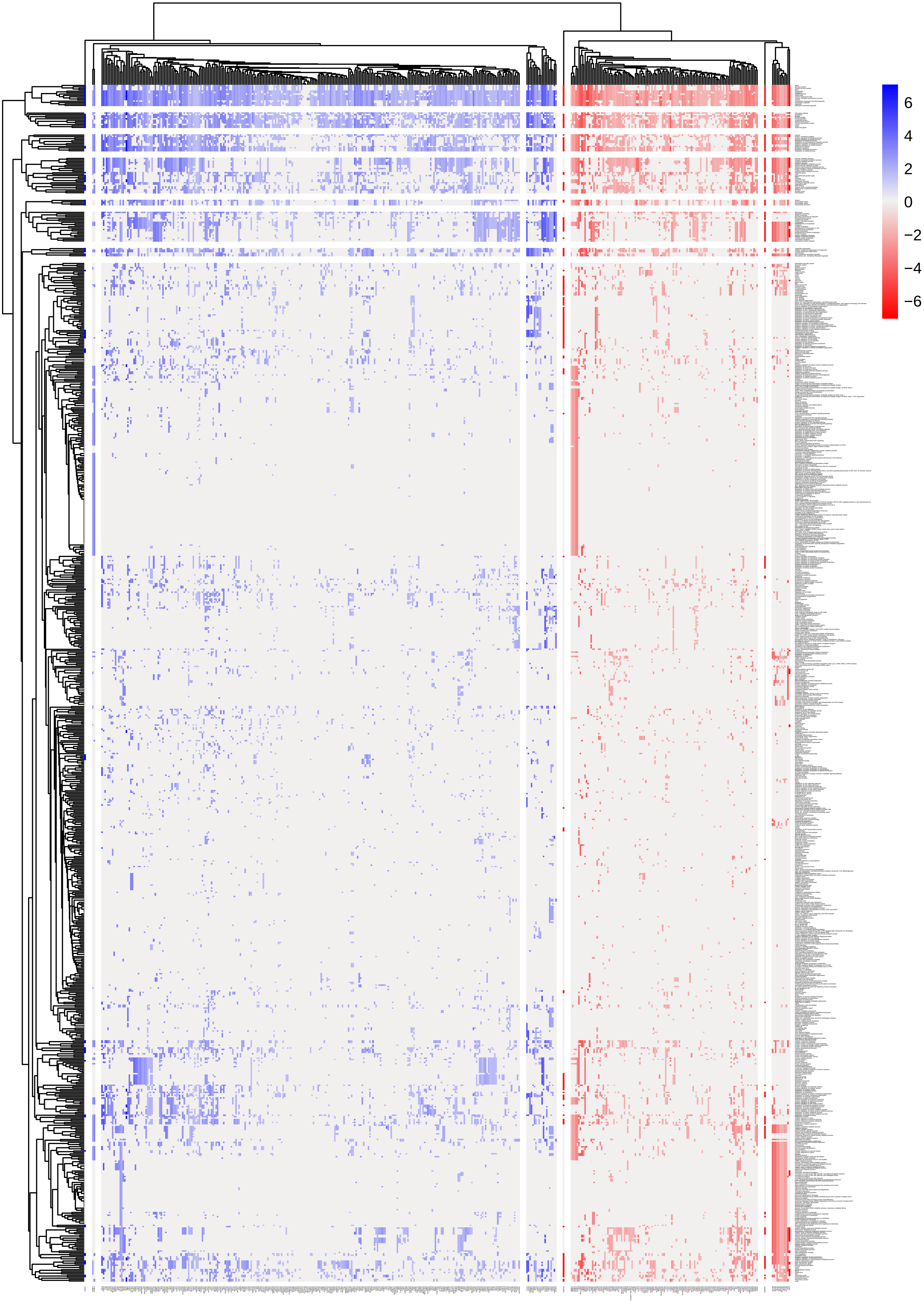
6
4
2
0
−2
−4
−6

**Figure S1** Heatmap showing all significant proteins, fold change and their correlation with the string functional network analysis. Blue increase in abundance in 3D samples compared to 2D samples. Red decrease in abundance in 3D samples compared to 2D samples.
